# Impact of interprofessional student led health clinics for patients, students and educators: a scoping review

**DOI:** 10.1007/s10459-024-10342-2

**Published:** 2024-06-06

**Authors:** Janine Prestes Vargas, Moira Smith, Lucy Chipchase, Meg E. Morris

**Affiliations:** 1 School of Allied Health, Human Services and Sport, La Trobe University, Bundoora, VIC 3086 Australia; 2 College of Healthcare Sciences, Department of Physiotherapy, James Cook University, Townsville, QLD 4814 Australia; 3 College of Nursing and Health Sciences, Flinders University, Adelaide, SA 5042 Australia; 4 Victorian Rehabilitation Centre, Glen Waverley, and ARCH and CERI La Trobe University, Bundoora, VIC 3086 Australia

**Keywords:** Clinical education, Interprofessional, Clinic, Student, Health education, Health professional

## Abstract

*Background*: Although interprofessional student led health clinics have been implemented worldwide, the impact of this model await confirmation. *Objectives*: To conduct a critical analysis of the literature on interprofessional student led clinics, and the views of stakeholders on feasibility and the barriers and facilitators to implementation. *Design*: A scoping review, evidence synthesis and quality appraisal were conducted using PRISMA Scr. Eight databases were searched from 2003 to 2023: Medline (Ovid), Embase (Ovid), CINAHL (EBSCO), Cochrane, Scopus, ERIC, Web of Science and Informit Health Collection. A qualitative descriptive approach was used to analyse data from patients, students and educators and inductive thematic analysis identified emergent themes. *Results*: Forty-six studies were included from a yield of 3140 publications. A key theme to emerge was that patients perceived improvements in their health and wellbeing and valued gaining health information from student led clinics. Student experiences were mostly positive although some found it challenging to work in interprofessional teams and roles were not always clear. The clinics enabled students to improve communication skills and autonomy. Clinical educators reported that students benefited from experiential learning within an interprofessional context. The clinics were feasible, provided that sufficient funding, infrastructure, staff and resources were available. Barriers to implementation included lack of funding, excessive waiting times and non-aligned student timetables. Pre-clinic orientation and support from on-site clinical educators facilitated implementation. *Conclusions*: Despite some challenges with implementing interprofessional student led clinics, they can have a positive impact on student learning and patient experiences.

**Supplementary Information:**

The online version contains supplementary material available at 10.1007/s10459-024-10342-2.

## Introduction

Interprofessional student led health clinics (SLC) have been implemented worldwide using different models, based on the healthcare needs of patients (Stetten et al., 2019) and available infrastructure, clinical supervision and resources (Buckley et al., 2014; Haggarty & Dalcin, 2014; Sick et al., 2014). Services are delivered by students from two or more health or social care disciplines, supervised by a registered health professional in a community-based health or social care setting (Briggs & Fronek, 2020; Lie et al., 2016). Interprofessional student led clinics have been argued to benefit students and patients (Broman et al., 2022; Schutte et al., 2015; Tokolahi et al., 2021). They often involve a combination of medical, nursing and allied health students (Horbal et al., 2019; Schmitt et al., 2013) and sometimes include non-health trainees such as interpreters (Davis, 2021), engineering students (Hayward et al., 2016) or law students (Rock et al., 2014). Students can be involved in clinics as volunteers or through university clinical placements (Forbes et al., 2021; White et al., 2018). A wide range of student led clinics have been reported, including student led, student run, student-delivered, student-assisted, student-initiated, student-facilitated, student-implemented, and student focussed service-learning clinics conducted as part of clinical education placements (Huang et al., 2021; Leung et al., 2016, 2022; Wynne & Cooper, 2021).

Interprofessional student led clinics give students the opportunity to learn with, from and about each other (Hopkins et al., 2022; World Health Organization, 2010). Students have reported increased ownership and responsibility for client care when participating in this type of clinical education (Schutte et al., 2015). There is also preliminary evidence of improved collaborative skills (Hopkins et al., 2022; Wilson et al., 2023), improved role clarity (Horbal et al., 2019), better understanding of patient-centred care (Huang et al., 2021) and an appreciation for working with disadvantaged people (Sick et al., 2017; Wilson et al., 2023). Briggs and Fronek (2020) reviewed student experiences of student led clinics, finding them to facilitate teamwork, a finding shared by Timm and colleagues (2021).

Student run free clinics date back to the 1960s and began in the USA as altruistic student enterprises to serve minority groups (Simpson & Long, 2007). Some clinics enabled access to healthcare for people who could not afford private health insurance (Palma et al., 2020; Stuhlmiller & Tolchard, 2015). They included low-income individuals, homeless people, immigrants, refugees and others (McElfish et al., 2018; Ng & Hu, 2017; Sick et al., 2017). Student clinics sometimes benefit people living in rural areas (Spencer et al., 2015; Stuhlmiller & Tolchard, 2015) and older people (Fung et al., 2022; Kent et al., 2016). They can provide low cost health services (Danhausen et al., 2015; Kent & Keating, 2013) and be effective at improving the management of chronic conditions (Gustafsson et al., 2016; Suen et al., 2020). Patients have reported benefits from the health knowledge gained (Burgess & Roberts, 2022; Ng et al., 2020), improved lifestyle and self-efficacy (Stuhlmiller & Tolchard, 2018) and training of students (Isaacson et al., 2014; Meuser et al., 2022). Clinical educators noted that pre-clinical training is usually discipline specific (Hall et al., 2012; VanKuiken et al., 2016), and interprofessional clinics afford greater preparedness for multi-disciplinary practice (Lestari et al., 2016; van Diggele et al., 2020).

With the rapidly growing literature on this topic, there is a need to synthesise recent findings on patient, student and clinical educator views on student led interprofessional clinics. The aim of this review was to address this gap and to understand the feasibility and barriers and facilitators to implementation of this model of health professional education.

## Methods

### Protocol and registration

This scoping review was completed according to the Preferred Reporting Items for Systematic Reviews and Meta-Analyses Extension for Scoping Reviews (PRISMA-ScR) (Tricco et al., 2018). It was based on the five stages of the methodological framework by Arksey and O’Malley (2005), later refined by Levac and colleagues (2010). Scoping reviews map, summarize and disseminate the available evidence on a topic (Munn et al., 2022). They also help to evaluate the type, range and extent of research already published (Arksey & O’Malley, 2005; Peters et al., 2020). The review was registered a-priori in Open Science Framework (10.17605/OSF.IO/5VBDZ).

### Identifying relevant studies

#### Eligibility criteria

Studies were included if they: (i) described interprofessional health services delivered to patients by students of at least two healthcare disciplines. Students of one discipline had to work together with students of another discipline. (ii) reported outcomes for patients, students and clinical educators and (iii) were peer-reviewed. A clinical educator was defined as a registered professional, affiliated with an educational institution or healthcare facility who was responsible for supervising or educating students. Reasons for excluding studies were: (i) no patient outcomes reported, (ii) not in English, (iii) publication older than 20 years, (iv) intervention delivered by a clinician not a student, (v) students not enrolled in higher education, (vi) opinion piece, thesis or review, or (vii) full text not available.

#### Search

A search strategy was created using both Medical Subject Headings (MeSH) terms and free text terms, available in Supplementary Material 1. This process was guided by a university librarian and was iterative and cross checked with key relevant literature in the topic. The key search concepts were: (i) student led health clinics and (ii) interprofessional structure of service delivery (two or more health disciplines working together). The search strategy was initially developed for Medline (Ovid) and subsequently adapted to each of the following databases: Embase (Ovid), CINAHL (EBSCO), Cochrane, Scopus, ERIC, Web of Science and Informit Health Collection. Additional studies were hand searched in reference lists of included studies and existing systematic reviews relevant to this field of research. A simple search was also conducted in the Physiotherapy Evidence Database (PEDro) to screen for relevant studies not picked up elsewhere.

#### Study selection

Covidence®, a web-based software to manage reviews, was used (Covidence systematic review software). In the first stage of screening, duplicates were removed, all titles and abstracts were screened by two blinded reviewers (JPV, MS), followed by full text screening against the inclusion and exclusion criteria. Disagreements were resolved by consensus with a third reviewer (MEM).

### Data charting

Data from each article were extracted using the Covidence® platform, then transferred to a database in Excel. A data extraction chart was created to collect specific information on the characteristics of interprofessional student led health clinics. We extracted data for the following variables: author, year of publication, country, study design, study aims, clinic or project name, key term used to describe the service (student led /run /developed /assisted /service learning), target population, number of patients, setting, disciplines and numbers of students and supervisors, types of intervention delivered by students, amount and mode of supervision, period of data collection, duration and type of student involvement in the clinic (placement or volunteer), method of collaboration between students (teams, planning and debrief meetings, group preparation or delivery of educational content) outcome measures, results, barriers and facilitators, and major findings. Feasibility data were extracted from each article, pertaining to (i) technical resources, equipment, set up, environment (ii) cost/ benefit (iii) organisational structure, processes, staffing, skills (iv) marketing of clinic services. We also analysed the barriers and facilitators to implementation of interprofessional student led clinics.

### Collating, summarising and reporting the results

Quantitative data were tabulated (Supplementary Material 2) and summarized to present an overview of student clinics identified in the review, including the geographical location, design, healthcare setting, disciplines, numbers of participants, target clientele and type of intervention. A qualitative descriptive approach was employed to analyse the qualitative data on three primary populations of interest: patients, students and clinical educators (Stanley, 2014). Each of the three datasets were coded separately and inductive thematic analysis was used to identify emergent themes from the bottom up (Braun & Clarke, 2006). Codes and themes were checked and verified by three reviewers (JPV, MEM, MS).

### Quality appraisal

Critical appraisal of the included studies was conducted using the Mixed Methods Assessment Tool (MMAT) (Hong et al., 2018). The MMAT assesses methodological quality for categories: (i) qualitative (ii) quantitative randomised controlled trial (RCT) (iii) quantitative non-RCT (iv) quantitative descriptive (v) mixed methods. The quality appraisal was completed by two reviewers (JPV, CT) and consensus was provided by a third reviewer (MEM) where required. We included an overall quality rating by adding the number of items marked ‘yes’ (i.e. total of ‘1/yes’ scores for each category; response range = 0–5) (Supplementary Material 3).

## Results

### Study characteristics

**Fig. 1:**
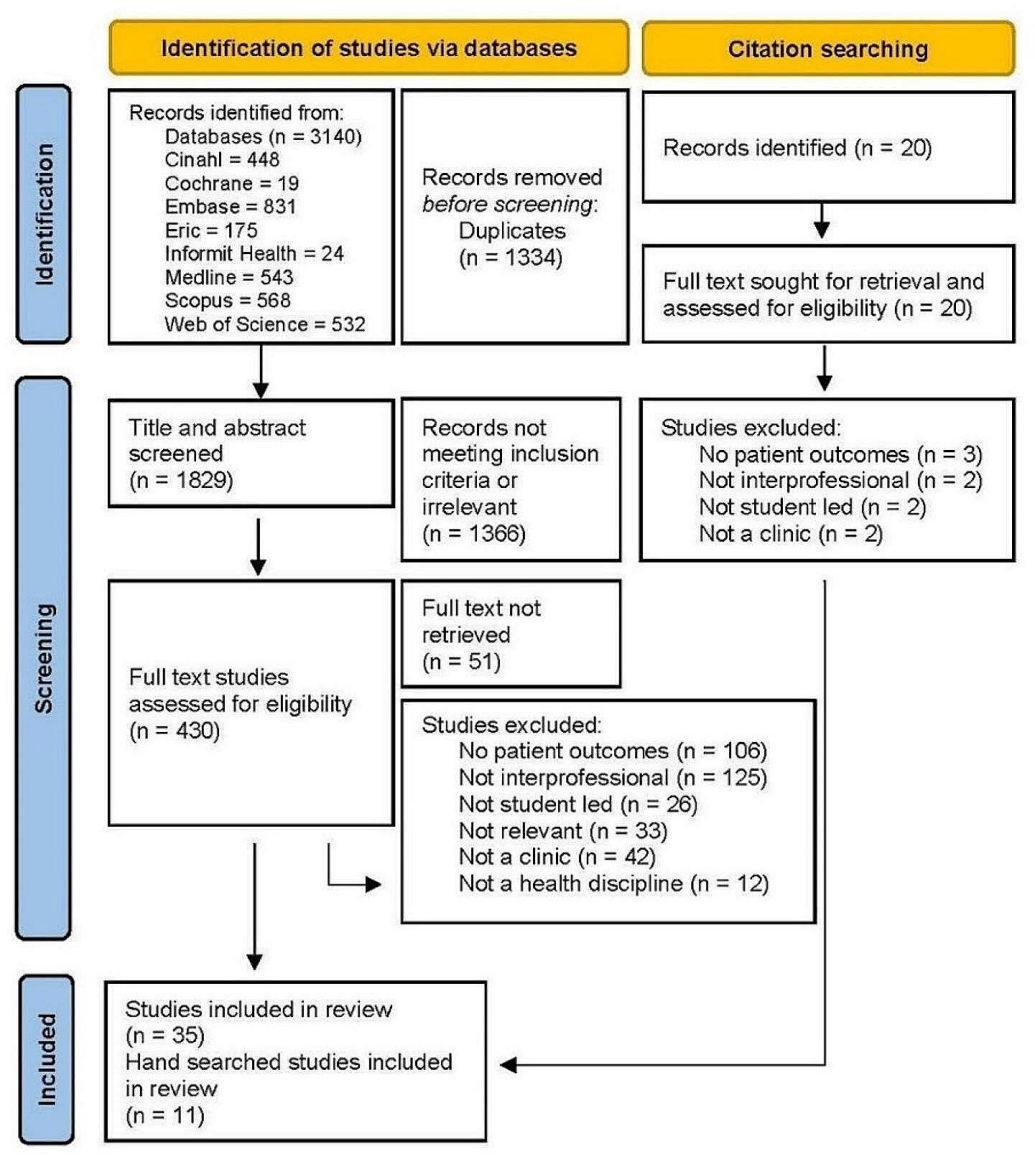
PRISMA flow diagram of study results

The PRISMA flow chart demonstrates the flow of studies (Fig. 1). Database searches yielded a total of 3140 citations. Citation searching yielded 20 results. After duplicates were removed, 430 full text studies were analysed, and a further 395 were excluded. Eleven of the 20 hand searched studies were excluded. Systematic reviews were set aside for referencing yet excluded from the searches due to duplication of included studies. A total of 46 studies were included in this scoping review and were from the USA, Australia, Canada, the Netherlands, Sweden, South Africa, China, Hong Kong and Singapore.

Most studies were conducted in primary healthcare settings (*n* = 25) (Supplementary Material 2). Other SLC took place in hospitals (Bird et al., 2022; Burgess & Roberts, 2022; Janson et al., 2009; Meek et al., 2013; Reumerman et al., 2021, 2022; Sultan et al., 2022), at home visits (Bird et al., 2022; Liang En et al., 2011; Ng et al., 2020; Rock et al., 2014; Sarovich et al., 2022; Seymour & Cannon, 2010), via telehealth (Fung et al., 2022; Henderson-Kalb et al., 2022; Leung et al., 2012; Meuser et al., 2022; Walker et al., 2022), in churches (Gortney et al., 2018; Johnston et al., 2019, 2020), in nursing homes (Dacey et al., 2010; Krout et al., 2010) mobile clinics (Palma et al., 2020), community housing (Busen, 2014; Howell et al., 2021; Krout et al., 2010), detention centres (Shekar et al., 2020), public spaces (Bird et al., 2022; Brown et al., 2015; Sarovich et al., 2022) and pre-schools (Sargison et al., 2021). Nine studies were conducted in more than one setting (Bird et al., 2022; Brown et al., 2015; Busen, 2014; Dacey et al., 2010; Henderson-Kalb et al., 2022; Krout et al., 2010; Leung et al., 2012; Liang En et al., 2011; Sarovich et al., 2022) (Supplementary Material 2).

As shown by Supplementary Material 2, interprofessional education was implemented before, during and after students delivered health services. Students from two or more disciplines worked together or participated in one or more of the following activities: preparing patient educational material, designing exercise classes, attending lectures, attending practical tutorials or role-play sessions, presenting about a topic to peers and faculty, orientation, shadowing senior students or being mentored by them, conducting patient assessments, delivering group or individual treatment sessions, meeting to plan and evaluate sessions, and co-designing the clinic with faculty staff.

Services offered to patients at interprofessional SLC included individual therapy, group therapy, vaccinations, client assessments, health screening, education, medication review, welfare assistance, equipment prescription and health referrals (Supplementary Material 2).

### Methodological quality

Quality appraisal results are presented in Supplementary Material 3. Studies used a variety of designs, including qualitative (Bird et al., 2022; Burgess & Roberts, 2022; Garavelis et al., 2023; Henderson-Kalb et al., 2022; Johnston et al., 2020; Meuser et al., 2022; Sargison et al., 2021; Sarovich., et al. 2022; Walker et al., 2022), quantitative RCT (Reumerman et al., 2022; Rock et al., 2014; Sultan et al., 2022), quantitative non-RCT (Brown et al., 2015; Janson et al., 2009; Lawrence et al., 2015; Ouyang et al., 2013; Virtue et al., 2018), quantitative descriptive (Asanad et al., 2018; Brown et al., 2021; Danhausen et al., 2015; Gortney et al., 2018; Hu & Leung, 2016; Johnston et al., 2019; Kahkoska et al., 2018; Kent & Keating, 2013; Kent et al., 2016; Krout et al., 2010; Ng et al., 2020; Palma et al., 2020; Peluso et al., 2014; Rowe et al., 2021; Shekar et al., 2020; Sheu et al., 2010), and mixed methods (Beckman et al., 2022; Busen, 2014; Dacey et al., 2010; Felder-Heim & Mader, 2020; Fröberg et al., 2018; Fung et al., 2022; Howell et al., 2021; Leung et al., 2012; Liang En et al., 2011; Meek et al., 2013; Reumerman et al., 2021; Sealey et al., 2017; Seymour & Cannon, 2010).

Twenty-one (46%) studies satisfied all five appraisal criteria of the appropriate category of the Mixed Methods Appraisal Tool, with the remaining studies satisfying four appraisal criteria (13 studies, 28%), three (6 studies, 13%), two (3 studies, 6%) and one appraisal criterion (2 studies, 4%). One study scored zero. The mean number of quality indicators with satisfied criteria was 4 (range 0 to 5) (Supplementary Material 3). Quantitative non-RCT studies received the highest quality ratings within the category (i.e., a score of four or five out of five) (4 out of 5 studies, 80%), followed by qualitative designs (7 out of 9 studies, 78%), mixed methods (10 out of 13 studies, 77%), quantitative descriptive (12 out of 16 studies, 75%) and quantitative RCTs (2 out of 3 studies, 67%). This reflects the use of mostly sound methodological approaches, interventions and interpretation of results based on data collected. Importantly, data from controlled (Brown et al., 2015; Janson et al., 2009; Lawrence et al., 2015; Ouyang et al., 2013; Reumerman et al., 2022; Rock et al., 2014; Sultan et al., 2022; Virtue et al., 2018) and less rigorous designs pointed in the same direction, with mostly positive patient and student outcomes (Supplementary Material 4 and 5).

### Outcome measures

#### Patient outcome measures

As seen in Supplementary Material 4, all studies reported data for patient outcomes. Patient outcomes were determined from self-reported surveys and questionnaires (27 studies), interviews and focus groups (8 studies) and clinical tests (14 studies). Twenty-six surveys or questionnaires were used to investigate patient outcomes and 14 of these were validated tools (Supplementary Material 4). Self-reported measures related to health knowledge, satisfaction with the interventions received or experiences of attending student clinics (Asanad et al., 2018; Beckman et al., 2022; Busen, 2014; Dacey et al., 2010; Fröberg et al., 2018; Fung et al., 2022; Gortney et al., 2018; Henderson-Kalb et al., 2022; Howell et al., 2021; Kent & Keating, 2013; Kent et al., 2016; Krout et al., 2010; Lawrence et al., 2015; Leung et al., 2012; Liang En et al., 2011; Meek et al., 2013; Meuser et al., 2022; Ng et al., 2020; Ouyang et al., 2013; Palma et al., 2020; Rock et al., 2014; Rowe et al., 2021; Sealey et al., 2017; Seymour & Cannon, 2010; Shekar et al., 2020; Virtue et al., 2018; Walker et al., 2022). The findings are presented in the thematic analysis below.

Patient clinical outcomes often pertained to body weight (Brown et al., 2015; Sealey et al., 2017), blood pressure/ heart rate (Felder-Heim & Mader, 2020; Janson et al., 2009; Johnston et al., 2019; Sealey et al., 2017), chronic disease status (Kahkoska et al., 2018; Janson et al., 2009), medication (Johnston et al., 2019; Reumerman et al., 2021; Sultan et al., 2022) or vaccination (Brown et al., 2021; Sheu et al., 2010). Two functional outcome measures were reported: (i) number of steps determined from a pedometer (Sealey et al., 2017) (ii) scores on the Physical Performance Test (Seymour & Cannon, 2010).

### Student Outcome measures

Eighteen studies reported student outcomes (Supplementary Material 5) using validated self-reported measures such as surveys and questionnaires (Dacey et al., 2010; Fröberg et al., 2018; Howell et al., 2021; Janson et al., 2009; Liang En et al., 2011; Ng et al., 2020; Sealey et al., 2017), non-validated self-reports (Busen, 2014; Fung et al., 2022; Krout et al., 2010; Liang En et al., 2011; Meuser et al., 2022; Reumerman et al., 2021; Sealey et al., 2017; Seymour & Cannon, 2010), and interviews or focus groups (Beckman et al., 2022; Bird et al., 2022; Sealey et al., 2017; Walker et al., 2022). The most common validated measures used were the Readiness for Interprofessional Learning Scale and the Fund for the Improvement of Post-Secondary Education Survey Instrument (Supplementary Material 5).

### Clinical Educator Outcome measures

Only three investigations reported clinical educator outcomes. Data collection methods included interviews (Beckman et al., 2022; Bird et al., 2022; Fröberg et al., 2018) and a non-validated self-reported survey (Beckman et al., 2022).

### Thematic analysis

#### Patient views on interprofessional student led clinics

Our qualitative analyses of patient views yielded three key themes about interprofessional student led clinics (Table 1 and Supplementary Material 4).

**Table 1:** Patient views on interprofessional student led clinics

Themes	Subthemes
**A. Improving personal health and health knowledge**	• improving understanding about health condition • improving self-monitoring • implementing health behaviour change • managing chronic diseases through health screening, medication, exercise therapies and referrals • improving lifestyle through exercise, diet, smoking, alcohol intake, sleep and mental health • fewer hospital admissions and emergency department visits
**B. Acknowledging services received from interprofessional student teams**	• feeling listened to, understood, consulted and respected • valuing the social contact • longer appointment times • some difficulties with student rapport building and wait times • clean, safe and friendly environment
**C. Improving access to healthcare for underserved people**	• discovering the health needs of underserved people • acknowledging poor availability of health services • free clinics often the only source of healthcare

#### Improving personal health and health knowledge

Patients reported satisfaction with the knowledge and information gained from attending student led interprofessional clinics, with most reporting improved understanding of their own health and wellbeing (Burgess & Roberts, 2022; Busen, 2014; Danhausen et al., 2015; Fung et al., 2022; Garavelis et al., 2023; Gortney et al., 2018; Howell et al., 2021; Kent et al., 2016; Ng et al., 2020). They appreciated students giving clear explanations and advice on topics such as exercise (Howell et al., 2021), managing COVID19 (Fröberg et al., 2018), tobacco cessation (Virtue et al., 2018), prenatal care (Danhausen et al., 2015), personal hygiene (Gortney et al., 2018; Sealey et al., 2017) and how to minimise hospital readmissions (Seymour & Cannon, 2010). Having their questions answered was a key reason for strong patient satisfaction (Busen, 2014; Kent & Keating, 2013; Lawrence et al., 2015; Palma et al., 2020) (Supplementary Material 4).

Patients also reported learning about how to monitor their own health and wellbeing (Supplementary Material 4) (Busen, 2014; Dacey et al., 2010; Danhausen et al., 2015; Felder-Heim & Mader, 2020; Leung et al., 2012; Liang En et al., 2011; Rock et al., 2014; Sealey et al., 2017). They valued the ways in which SLC fostered health promotion (Dacey et al., 2010; Howell et al., 2021; Kent & Keating, 2013; Leung et al., 2012; Reumerman et al., 2021; Sealey et al., 2017; Seymour & Cannon, 2010), healthy aging (Dacey et al., 2010; Howell et al., 2021; Sarovich et al., 2022) and healthy lifestyle choices (Howell et al., 2021; Leung et al., 2012; Sealey et al., 2017). This empowered patients to change their health behaviours and to recognise when their health was changing (Burgess & Roberts, 2022; Busen, 2014).

*“It has woke [sic] me up and think that actually each one of these things is part of my health and I need to update it all the time, I need to stay on top of my exercise, I need to stay on top of my healthy eating, I need to go to the dentist, I need to get my prostate checked, I need to do all of these to keep rolling forward.”* (Sealey et al., 2017, pg. 1137).

Patients advised health benefits from frequent health screening (Danhausen et al., 2015; Felder-Heim & Mader, 2020; Leung et al., 2012; Liang En et al., 2011; Rock et al., 2014). Many appreciated person-centred, multi-disciplinary care (Burgess & Roberts, 2022; Liang En et al., 2011; Sargison et al., 2021). Many patients were diagnosed with new conditions or received treatment for existing conditions such as cardiovascular disease (Gortney et al., 2018; Johnston et al., 2019, 2020), respiratory infections (Danhausen et al., 2015; Gortney et al., 2018; Johnston et al., 2019, 2020; Rock et al., 2014), infectious diseases (Danhausen et al., 2015; Johnston et al., 2019, 2020), or dental problems (Busen, 2014; Johnston et al., 2020) (Supplementary Material 4).

As shown in Supplementary Material 4, several authors reported improvements in patient bodyweight (Brown et al., 2015; Leung et al., 2012), blood pressure (Felder-Heim & Mader, 2020; Rowe et al., 2021), cholesterol levels (Janson et al., 2009), smoking (Virtue et al., 2018), diabetes (Kahkoska et al., 2018; Rowe et al., 2021), and medication adherence (Leung et al., 2012; Rock et al., 2014). Many patients had their medications reviewed, changed or prescribed in the student led clinics (Kent & Keating, 2013; Kent et al., 2016; Leung et al., 2012; Liang En et al., 2011; Ng et al., 2020; Peluso et al., 2014; Reumerman et al., 2021, 2022; Rowe et al., 2021; Sultan et al., 2022; Virtue et al., 2018). Some received medications or vaccinations for free or at a low-cost (Asanad et al., 2018; Brown et al., 2021; Johnston et al., 2019; Ouyang et al., 2013; Palma et al., 2020; Sheu et al., 2010). Referrals to other services, such as emergency departments and community health centres, were made by many clinics (Asanad et al., 2018; Danhausen et al., 2015; Gortney et al., 2018; Hu & Leung, 2016; Janson et al., 2009; Johnston et al., 2019, 2020; Kent & Keating, 2013; Kent et al., 2016). Student led clinics reduced hospital admissions and emergency department use for some patients (Janson et al., 2009; Rock et al., 2014; Rowe et al., 2021).

#### Patients valued services received from interprofessional student teams

Supplementary Material 4 shows that most patients reported positive experiences with SLC (Bird et al., 2022; Fung et al., 2022; Garavelis et al., 2023; Gortney et al., 2018; Henderson-Kalb et al., 2022; Kent et al., 2016; Lawrence et al., 2015; Liang En et al., 2011; Sargison et al., 2021; Sarovich et al., 2022). They felt respected, understood and listened to by students (Fröberg et al., 2018; Garavelis et al., 2023; Howell et al., 2021; Johnston et al., 2020; Kent et al., 2016; Krout et al., 2010; Liang En et al., 2011; Sargison et al., 2021; Sarovich et al., 2022).

*“The experience was very good, very accurate, and very attentive, they knew what they were doing, there was little confusion between what was happening…the reporting and the questioning was done very thoroughly and covered a lot of stuff and they listened and understood what I was explaining”* (Garavelis et al., 2023, pg. 6).

The value of social interactions with students, peers and clinical supervisors was also noted by patients (Burgess & Roberts, 2022; Fung et al., 2022; Henderson-Kalb et al., 2022; Howell et al., 2021; Krout et al., 2010; Ng et al., 2020; Sarovich et al., 2022) (Supplementary Material 2). For some it was the most enjoyable aspect of participating (Seymour & Cannon, 2010).

*“The interaction with my peers [was most valuable], because I learned that I am loved and wanted.”* (Howell et al., 2021, pg. 259).

In general, patients enjoyed the contact with young people and meeting others with similar health conditions (Fung et al., 2022; Howell et al., 2021). Participants at a remote indigenous community valued the potential to share cultural knowledge with future generations through their connections with students (Sarovich et al., 2022).

*“When you guys learn [here, you] also learn a different culture too, to pass on to like, the next community go and do this with the Elders…”* (Sarovich et al., 2022, pg. 5).

Longer appointment times were appreciated (Fröberg et al., 2018; Johnston et al., 2020; Kent & Keating, 2013; Kent et al., 2016; Ng et al., 2020), although some patients commented on home visits being too long or too short (Ng et al., 2020). Excessive waiting times were also noted by some patients (Asanad et al., 2018; Lawrence et al., 2015).

#### Improving access to healthcare for underserved people

Student-run free clinics often provided services for patients who were unable to access healthcare from government agencies or via other means (Asanad et al., 2018; Brown et al., 2021; Henderson-Kalb et al., 2022; Johnston et al., 2020) (Supplementary Material 4). This particularly applied to homeless people, who struggled to maintain a healthy lifestyle when living on the streets (Johnston et al., 2020). Many experienced theft, poor hygiene, exposure to the elements and diseases (Asanad et al., 2018; Johnston et al., 2020).

*“During winter it’s whereby we experience a high number of death rates on the street, due to the fact that people, they don’t know their health state.”* (Johnston et al., 2020, pg. 4).

Some patients reported barriers to access healthcare, such as the cost of transport and comorbidities (Johnston et al., 2019; Peluso et al., 2014). Sometimes health insurance was not affordable or available, due to immigration status or the country of residence (Asanad et al., 2018; Brown et al., 2021; Peluso et al., 2014). Student clinics were the main source of healthcare for many underserved groups and helped to address some of these issues (Asanad et al., 2018; Brown et al., 2021; Palma et al., 2020; Sarovich et al., 2022). Some homeless people reported a preference for services to be more frequent, such as dental, social and psychology services.

#### Student outcomes in interprofessional student led clinics

As shown in Table 2 and Supplementary Material 5, six main themes emerged.

**Table 2:** Student outcomes

Themes	Subthemes
**A. Improving teamwork**	• learning with and from each other • increasing respect towards other professions • teamwork enabling development of leadership skills
**B. Acquiring and applying knowledge**	• understanding own role and role of others • understanding care needs of older people • practicing patient-centred care and the holistic approach • learning about practice management including service structure and delivery
**C. Improving communication skills**	• increasing collaboration with other students through communication • learning to adapt communication with patients, educators and other students • improving public speaking and presentation skills
**D. Valuing participation in student teams**	• enjoying the educational environment • increasing responsibility, autonomy, confidence and readiness for practice • valuing the individualised care provided to patients • valuing the relationship, support and feedback from supervisors
**E. Increasing social awareness**	• changing perceptions towards older people • being aware of needs of disadvantaged groups
**F. Addressing challenges of interprofessional student led clinics**	• being unclear about the role of other professions • feeling unprepared for interprofessional practice • some students feeling stressed with the increased responsibility • experiencing challenges in telehealth • coaching patients with multiple health concerns was difficult

#### Improving teamwork

Students valued the opportunity to work in teams (Henderson-Kalb et al., 2022; Howell et al., 2021; Krout et al., 2010; Liang En et al., 2011) (Supplementary Material 5). Interprofessional teams helped them to develop mutual respect (Bird et al., 2022; Dacey et al., 2010), confidence and preparedness for future clinical practice (Beckman et al., 2022; Ng et al., 2020; Reumerman et al., 2021). Students who held leadership positions gained a better understanding of collaborative practice (Liang En et al., 2011). The importance of input from a range of disciplines in patient care was noted:

*“The knowledge from psychology is a little bit different to counselling, and then you have… social work [with] more… resources, and I think it’s just really powerful having the combination of the different professions.”* (Beckman et al., 2022, pg. 99).

#### Acquiring and applying knowledge

Students reported gaining knowledge in four key areas: scope of practice, healthcare needs of older people, patient-centred care and practice management and administration. Improved knowledge about their scope of practice and the role of other professionals in the team was particularly valued (Bird et al., 2022; Busen, 2014; Howell et al., 2021; Reumerman et al., 2021; Sealey et al., 2017; Seymour & Cannon, 2010). Some students realised how little they knew about the role of others, as well as how this changed through participation:

*“I looked up the scope of practice for nurse practitioners…it was interesting to read what my scope would be and try and explain it to other disciplines. After class…it was interesting to hear how other professions viewed us.”* (Busen, 2014, pg. 364).

Knowledge acquired from working with older adults was noted. Some students felt that they acquired an improved understanding of the needs of vulnerable older people, especially when they had limited healthcare resources in the community (Dacey et al., 2010; Fung et al., 2022; Howell et al., 2021; Ng et al., 2020; Seymour & Cannon, 2010).

*“I have learned that seniors do want to be actively engaged, and appreciate the opportunity to learn new things that will benefit them…. Working with seniors is a unique challenge, because it can be difficult to look at things from their perspective….”* (Dacey et al., 2010, pg. 698).

Knowledge of patient-centred care enabled some students to identify the impact of health conditions on the ability of people to perform activities of daily living (Bird et al., 2022; Seymour & Cannon, 2010). It was reported that real-life clinical experiences acquired in an interprofessional setting helped them to develop a holistic approach to healthcare (Bird et al., 2022; Dacey et al., 2010; Fröberg et al., 2018).

Many students participated in free clinics delivered by an interprofessional student council, where student leaders oversaw the logistical and administrative tasks of the clinic (Asanad et al., 2018; Brown et al., 2021; Busen, 2014; Danhausen et al., 2015; Gortney et al., 2018; Liang En et al., 2011; Ouyang et al., 2013; Palma et al., 2020; Peluso et al., 2014; Reumerman et al., 2022; Sheu et al., 2010; Sultan et al., 2022). This assisted them to develop leadership skills, and experience in service design and service implementation (Danhausen et al., 2015; Liang En et al., 2011). Students also learned about administration and clinic management:

*“NHS is the best volunteer program out there because it is initiated by students, done by students, and supported by everyone else. I was placed in charge of a team, and it was wonderful to have the opportunity to guide my juniors along, as well as take charge of handling the patient’s care.”* (Liang En et al., 2011, pg. 834).

#### Improving communication skills

Most students reported improvements in their communication skills. Rapport building with patients, educators and other students also improved (Bird et al., 2022; Howell et al., 2021; Leung et al., 2012; Meuser et al., 2022) (Table 2 and Supplementary Material 5). This was particularly true when orientation was delivered prior to service (Bird et al., 2022; Meuser et al., 2022). Clinics delivered via telehealth helped students to develop their technology and management skills (Meuser et al., 2022; Walker et al., 2022). A number of students reported improvements in public speaking skills (Howell et al., 2021; Sealey et al., 2017). Improved communication was linked to service quality and efficiency:

*“…I learned how really utilizing all professions can improve patient outcomes. I also learned that through good communication skills and respect for all health care professions we can accomplish higher quality work in a timelier manner.”* (Dacey et al., 2010, pg. 698).

#### Valuing participation in student teams

Most students found that the interprofessional clinic environment provided a valuable team experience (Fröberg et al., 2018; Henderson-Kalb et al., 2022; Krout et al., 2010; Reumerman et al., 2021). Some saw direct relevance to the content about teamwork learned in class (Krout et al., 2010) and to future practice (Beckman et al., 2022). Support and trust gave them increased confidence and autonomy with teamwork and patient care (Bird et al., 2022; Dacey et al., 2010).

*“During this project I have learned many things about myself as well as my team members…. I have learned to become more adaptable which will help me to overcome obstacles in my profession. Also, I strongly believe that the key to success and being able to overcome any barrier we are presented with is the ability to stay confident and always remain optimistic. This class has given me the confidence and optimism necessary to overcome any problem”.* (Dacey et al., 2010. pg. 698)

#### Increasing social awareness

Many students gained more positive perceptions about working with older adults during the clinics (Table 2 and Supplementary Material 5). Some changed their negative preconceived ideas about the capabilities and interests of older people (Meuser et al., 2022; Ng et al., 2020).

*“This class will definitely be a memorable experience that will forever have an impact on both my personal and professional life. I can honestly say my learnings from this class have definitely changed my views toward the aging population.”* (Howell et al., 2021, pg. 260).

Working with underserved groups was also appreciated by students and benefited altruism (Busen, 2014; Howell et al., 2021; Liang En et al., 2011). The clinics facilitated cultural competence (Bird et al., 2022), especially when providing services to marginalised groups (Bird et al., 2022; Busen, 2014; Danhausen et al., 2015). Skills in identifying healthcare gaps were noted:

*“The NHS program is helpful in exposing us as students to the poor living conditions and health knowledge of residents in Taman Jurong. Hopefully it also helps the patients by picking up otherwise undetected diseases and reintegrating patients who have fallen out of the health care system.”* (Liang En et al., 2011, pg. 835).

#### Student views of challenges

Having a poor understanding of the role of other health professionals at the start of the clinical placement was challenging for some students (Beckman et al., 2022; Bird et al., 2022; Busen, 2014; Sealey et al., 2017) (Tables 2 and Supplementary Material 5). Several requested more orientation and role-play prior to the clinic (Beckman et al., 2022; Howell et al., 2021). A small number of difficulties were reported in telehealth clinics related to rapport building with patients (Fung et al., 2022; Henderson-Kalb et al., 2022; Leung et al., 2012; Walker et al., 2022). A number required more training in online service delivery:

*“So if it was to be a known—say telehealth placement—it would be interesting to learn some of those communication strategies that you always—at uni you’re learning face to face, you’re trying to learn how to build rapport and all of that physical, body language, stuff like that. So I think it could have been beneficial to know that going in to be a bit more prepared.” (*Walker et al., 2022, pg. 86).

#### Clinical educator views in interprofessional student led clinics

Three main themes emerged from qualitative analysis of clinical educator interviews (Table 3 and Supplementary Material 6).

**Table 3:** Themes arising from clinical educators

Themes	Subthemes
**A. Facilitating the clinical educator role**	• Affording students sufficient time for patient consultations, clinical educator supervision and feedback • Optimal modelling and communication improved student learning • Improving communication between students, educators, clinics and universities
**B. Understanding roles, responsibilities and value of interprofessional teams**	• Educators facilitating student autonomy and a consistent approach to supervision • Educators feeling equipped and confident to deliver authentic interprofessional education • Improving student interprofessional practice
**C. Identifying challenges in delivery of student led clinics**	• Educators feeling stressed by non-aligned student timetables, limited time with students, and lack of training in how to deliver interprofessional supervision

#### Facilitating the clinical educator role

The clinical educator role was found to be facilitated in interprofessional SLC by allocating sufficient time for clinical supervision (Table 3 and Supplementary Material 6):

*“I believe that I have more time to engage in my role… even if you always try to but it is… it is different. It is quieter and I have more time to engage in the subject, I have time to look things up before so that I know what they are studying and what they are supposed to focus on and so forth.”* (Fröberg et al., 2018, pg. 42).

Regular meetings with stakeholders and students also enabled collaboration (Bird et al., 2022; Fröberg et al., 2018) and educators saw modelling and communication as beneficial outcomes:

*“I will go in and have a chat, even if it’s just “Hey, this is what I’m thinking, what do you reckon?” I think modelling that behaviour is a great approach.”* (Beckman et al., 2022, pg. 101).

#### Understanding roles, responsibilities and value of interprofessional teams

Clinical educators noticed the value of interprofessional SLC in boosting student confidence to work within a team, mostly due to improved knowledge of their own scope of practice, the role of others and improved communication skills (Bird et al., 2022; Fröberg et al., 2018). Student-centred supervision gave students more independence and responsibilities with patients (Fröberg et al., 2018).

*“I think their professional growth was enormous…they really grew in their ability.”* (Bird et al., 2022, pg. 82).

With regards to interprofessional supervision training, SLC provided a conducive environment for supervision and afforded opportunities for student-centred feedback (Beckman et al., 2022; Fröberg et al., 2018).

*“I have to say that there is much less stress at the SRC…one gets to focus on the supervisor role.”* (Fröberg et al., 2018, pg. 42) (SRC = student run clinic).

The interprofessional setting promoted student learning (Beckman et al., 2022; Bird et al., 2022).

*“Because working in unison, apart from anything else, can help them understand the life of the people they were working with. And really shift their thinking from a disability or a therapy focus, to a community empowerment focus.”* (Bird et al., 2022, pg. 80).

#### Challenges in delivery

Educators identified some procedural issues in running the interprofessional clinics (Beckman et al., 2022; Fröberg et al., 2018) (Table 3 and Supplementary Material 6). Sometimes it was difficult to align the timetables of students from different professions, particularly when different groups of students required orientation on the same day (Beckman et al., 2022). A few lacked necessary training and needed further professional development and orientation prior to supervising students in the clinics (Fröberg et al., 2018). Supervisors noted that some students lacked awareness of their own professional culture, which may have been a barrier in developing strong connections with other students, staff and the community (Bird et al., 2022). Some supervisors acknowledged the balance between student autonomy and supervisor control was challenging (Fröberg et al., 2018).

*“Then again it’s about control… And I guess that you are different about that and I believe that me as a person, I like to be in control. So you really need to challenge yourself, and at the same time you need to find that balance. So that it is still patient safe and, well. That is it, the difficult part. And sometimes you are in a good flow and sometimes it is more difficult…”* (Fröberg et al., 2018, pg. 43).

#### Feasibility and implementation

Feasibility is summarised in Table 4. Clinics were viable provided that there was adequate patient access, technical resources, equipment, infrastructure and availability of well trained clinical education staff (Table 4). Dedicated clinic funding and marketing increased the feasibility of interprofessional student led clinics (Table 4).

**Table 4:** Key facilitators and barriers to implementation of interprofessional student led clinics

**Technical feasibility (resources, equipment, set up, environment)**	
*Facilitators*	*Barriers*
• Interprofessional education in a real clinic setting • Clinic located in the community • Use of online platforms for delivery of interprofessional education • All disciplines operating in the same physical space	• Small clinic spaces could not accommodate several disciplines • Non-permanent clinic sites
**Operational feasibility (organisational structure, processes, staffing, skills)**	
*Facilitators*	*Barriers*
• Student orientation to tasks prior to involvement • Consistent support from clinical educators • Co-design with stakeholders • Interprofessional education in classroom • Regular interprofessional meetings • Student council overseeing clinic operations • Consistent and clear clinic documentation • Guiding templates to assist new students and improve clinic flow • Centralised whiteboard to assist with clinic flow • Focus on one patient population • Focus on health promotion • Easy to understand patient educational content • Patient and family-centred approach • Extended appointment times	• Timetabling students from different professions • Lack of consistent university support and resources • Wait times • Students at different stages of learning • Large numbers of rotating students • Recruiting and scheduling volunteers • Lack of student training in delivering telehealth
**Financial feasibility (cost/benefit)**	
*Facilitators*	*Barriers*
• Clinic added to an existing service	• Insufficient funding
**Market feasibility**	
*Facilitators*	
• Ongoing marketing of clinic	

Thirty studies reported barriers and facilitators to student clinic operations (Asanad et al., 2018; Bird et al., 2022; Brown et al., 2021; Busen, 2014; Dacey et al., 2010; Danhausen et al., 2015; Felder-Heim & Mader, 2020; Fröberg et al., 2018; Fung et al., 2022; Gortney et al., 2018; Henderson-Kalb et al., 2022; Howell et al., 2021; Hu & Leung, 2016; Janson et al., 2009; Kahkoska et al., 2018; Kent et al., 2016; Krout et al., 2010; Leung et al., 2012; Meek et al., 2013; Ouyang et al., 2013; Palma et al., 2020; Peluso et al., 2014; Reumerman et al., 2022; Rowe et al., 2021; Sargison et al., 2021; Sarovich et al., 2022; Sealey et al., 2017; Shekar et al., 2020; Sheu et al., 2010; Walker et al., 2022) (Table 4).

Frequently cited facilitators were (i) delivery of student orientation prior to student clinics (Beckman et al., 2022; Bird et al., 2022; Danhausen et al., 2015; Howell et al., 2021; Lawrence et al., 2015; Meuser et al., 2022; Ouyang et al., 2013; Sargison et al., 2021) (ii) consistent and positive support from educators (Bird et al., 2022; Dacey et al., 2010; Fröberg et al., 2018; Sealey et al., 2017; Seymour & Cannon, 2010; Sheu et al., 2010; Walker et al., 2022) and (iii) use of existing clinics (Bird et al., 2022; Dacey et al., 2010; Danhausen et al., 2015; Fröberg et al., 2018; Janson et al., 2009; Reumerman et al., 2022).

Frequently cited barriers were difficulty timetabling students from different disciplines (Beckman et al., 2022; Busen, 2014; Dacey et al., 2010; Danhausen et al., 2015; Gortney et al., 2018; Howell et al., 2021; Janson et al., 2009), low University support (Asanad et al., 2018; Beckman et al., 2022; Fröberg et al., 2018; Gortney et al., 2018; Henderson-Kalb et al., 2022; Krout et al., 2010; Meek et al., 2013; Rowe et al., 2021) and poor funding (Asanad et al., 2018; Danhausen et al., 2015; Leung et al., 2012; Sheu et al., 2010).

## Discussion

Student led clinics are increasingly being used to prepare students for the demands of future workforce requirements (Guitar & Connelly, 2021; Hopkins et al., 2022; Schutte et al., 2015). This review has generated new evidence that interprofessional SLC can have benefits for patients, students and clinical educators. Many patients reported a positive impact on health, knowledge, social interactions, or clinical outcomes. There was converging evidence that students felt better prepared for working in teams, taking responsibility for service delivery and providing client-centred care. Clinical educators highlighted the benefits of interprofessional supervision to student learning outcomes.

Over and above the previous reviews by Hopkins et al. (2022) and Schutte et al. (2015), our article adds new evidence on the benefits to patients participating in interprofessional SLC. Patient access to healthcare services was facilitated, particularly when clinics were delivered in the community (Bird et al., 2022; Brown et al., 2021; Howell et al., 2021), schools (Sargison et al., 2021), workplaces (Brown et al., 2015) and aged care (Busen, 2014). Extended time for consultations was valued by patients as it facilitated rapport building and enabled patients to feel listened to (Fröberg et al., 2018; Garavelis et al., 2023). This is in agreement with the work of Lemon et al. showing that consultation length plays an important role in patient satisfaction (Lemon & Smith, 2014). Improvements in patient personal health and health knowledge were also consistently demonstrated across the studies that we reviewed, in agreement with previous reports (Broman et al., 2022; Frakes et al., 2011; Suen et al., 2020).

Student learning outcomes were enhanced by interprofessional SLC, especially knowledge of professional roles, clinical skills and teamwork (Beckman et al., 2022; Bird et al., 2022; Dacey et al., 2010; Danhausen et al., 2015; Fung et al., 2022; Howell et al., 2021; Janson et al., 2009; Krout et al., 2010; Liang En et al., 2011; Ng et al., 2020; Reumerman et al., 2021; Sealey et al., 2017; Seymour & Cannon, 2010). These findings agree with Wilson et al. (2023) who reported that students enhanced their collaboration skills and empathy for vulnerable populations. Students also valued the feedback received from clinical educators (Fröberg et al., 2018; Sealey et al., 2017). This concurred with a report by Lie et al. (2016) who advocated for timely and relevant feedback to assist students to integrate their clinical skills. Students who received prior training on their role in the team had more positive experiences (Beckman et al., 2022; Bird et al., 2022).

Clinical educators noted an increased sense of student responsibility and preparedness for future practice (Beckman et al., 2022; Bird et al., 2022). The educators themselves gained knowledge and refined their collaborative skills (Fröberg et al., 2018). Similar findings were reported by Martin et al. (2022) who showed that clinical educators at rural SLC improved their interprofessional competencies. Some clinical educators felt unprepared for interprofessional supervision (Fröberg et al., 2018), a finding previously identified by Anderson et al. (2009). Improvements in confidence and skills were reported by educators who took part in training prior to involvement in interprofessional SLC (Anderson et al., 2009; Bird et al., 2022; Brown et al., 2015; Busen, 2014; Fung et al., 2022).

In agreement with other research, we found the benefits of interprofessional SLC also included a focus on health promotion (Dacey et al., 2010; Kent & Keating, 2013), genuine opportunities for interprofessional learning (Bird et al., 2022; Brown et al., 2021; Fung et al., 2022; Janson et al., 2009; Reumerman et al., 2022; Seymour & Cannon, 2010), better access to university resources (Asanad et al., 2018; Bird et al., 2022; Danhausen et al., 2015; Garavelis et al., 2023; Sarovich et al., 2022; Sealey et al., 2017) and opportunities for service co-design (Bird et al., 2022; Howell et al., 2021; Krout et al., 2010; Sarovich et al., 2022).

The feasibility of delivering interprofessional SLC was had some limitations and was affected by a range of issues. They were often difficult to timetable (Beckman et al., 2022; Busen, 2014; Dacey et al., 2010) or fund (Asanad et al., 2018; Danhausen et al., 2015; Kent et al., 2016). Some staff and students felt poorly prepared for this unique type of multi-disciplinary clinical placement (Beckman et al., 2022; Busen, 2014; Janson et al., 2009). Kent et al. (2014) observed that financial constraints could be a challenge, as well as staffing (Fröberg et al., 2018; Hu & Leung, 2016; Krout et al., 2010) and venue availability (Fröberg et al., 2018; Howell et al., 2021). Wait times were sometimes associated with dissatisfaction with free clinics (Asanad et al., 2018; Lawrence et al., 2015), a finding shared by others who sought effective strategies to improve efficiency (Lee et al., 2017; Stephens et al., 2020).

This scoping review has some limitations. Some studies were of modest methodological quality and most had comparatively small samples and non-randomized or non-comparative designs which increase the risk of bias (Supplementary Material 2 and 3). Broman et al. (2022) and Wilson et al. (2023) noted that heterogeneity and underpowered samples limit the generalisability of findings in this field. Less than half of studies reported student outcomes, and only a few collected data on clinical educator views. Around half of the studies with patient outcomes used self-reported methods which carry an increased risk of bias. Self-reported measures, while valuable for capturing individual experiences, carry social desirability bias, response bias, and recall bias (Althubaiti, 2016). This increased risk of bias underscores the importance of future investigations adopting robust study designs, with large sample sizes and diverse patient populations, using validated quantitative and qualitative outcome measures. It would also be helpful to compare the outcomes of student led clinics with professional-delivered interventions. Evaluation of long-term student outcomes upon entry to the workforce is also required.

In conclusion, student led health clinics can have positive impacts for student learning, especially in relation to interprofessional collaborative practice, skill development and readiness for entering the workforce. For patients, healthcare access can be facilitated and there are benefits for knowledge, health and wellbeing.

## Electronic supplementary material

Below is the link to the electronic supplementary material.

Supplementary Material 1

Supplementary Material 2

Supplementary Material 3

Supplementary Material 4

Supplementary Material 5

Supplementary Material 6

## Data Availability

All data sets discussed in the current review are available from the corresponding author on request.

## Declarations

This scoping review was registered in Open Science Framework, registration number osf.io/5vbdz.
