# Supplementary material for: Impact of interprofessional student led health clinics for patients, students and educators: a scoping review
Source: Adv Health Sci Educ Theory Pract. 2024 Jun 6;30(1):321–45. doi: 10.1007/s10459-024-10342-2 (PMC11925975; doi:10.1007/s10459-024-10342-2)
Supplement: Supplementary file 1 — Supplementary Material 1 [file 10459_2024_10342_MOESM1_ESM.pdf]

SUPPLEMENTARY MATERIAL 1: SEARCH STRATEGY

((student led OR student run OR student directed OR student managed OR student facilitated OR student developed OR student assisted) n3 (clinic* OR service* OR program* OR group* OR intervention*)) OR student initiat* OR ((“service learning”) n1 (clinic* OR course* OR program* OR project* OR practice* OR approach* OR method))

AND

Interprofessional OR “inter-professional” OR interdisciplinary OR “inter-disciplinary” OR multidisciplinary OR “multi-disciplinary” OR healthcare OR “health care” OR wellness OR wellbeing OR rehabilitation OR “tele-health” OR telerehabilitation OR “tele-rehabilitation” OR online

Article title: Impact of interprofessional student led health clinics for patients, students and educators: a scoping review
Journal name: Advances in Health Sciences Education
Author names: Janine Prestes Vargas, Moira Smith, Lucy Chipchase, Meg E. Morris
Affiliation of corresponding author: Victorian Rehabilitation Centre, Glen Waverley, and ARCH and CERI La Trobe University
Email of corresponding author: m.morris@latrobe.edu.au
