# Supplementary material for: Impact of interprofessional student led health clinics for patients, students and educators: a scoping review
Source: Adv Health Sci Educ Theory Pract. 2024 Jun 6;30(1):321–45. doi: 10.1007/s10459-024-10342-2 (PMC11925975; doi:10.1007/s10459-024-10342-2)
Supplement: Supplementary file 2 — Supplementary Material 2 [file 10459_2024_10342_MOESM2_ESM.pdf]

SUPPLEMENTARY MATERIAL 2: CHARACTERISTICS OF INTERPROFESSIONAL STUDENT LED CLINICS

Study	Country	Design	Setting	Disciplines	Clientele	Intervention
**Asanad 2018**	USA	Descriptive; Cross-sectional	Community clinic[F]	Medicine, Social Work, Public Health	n=194<br>Homeless people	Assessment<br>Therapy, individual<br>Education<br>Referrals<br>Welfare
**Beckman 2022**	Australia	Mixed methods	Community clinic[P]	Nursing, Physiotherapy, Occupational Therapy, Exercise Physiology, Dietetics, Social Work, Psychology, Counselling	n=23<br>Over 50s	Assessment<br>Therapy, individual and group<br>Education
**Bird 2022**	Australia	Qualitative	Community centre, home visit, hospital and public spaces[PH]	Occupational Therapy, Speech Pathology	n=11<br>Aboriginal elders and their family members	Assessment<br>Therapy, individual and group<br>Home modifications<br>Provision of aids and equipment<br>Education
**Brown 2015**	USA	Non-RCT	Community centre and patient's workplace[F]	Medicine, Nursing, Health Sciences	n=46<br>Low-income overweight and obese community residents	Student led vs professional led<br>Therapy, group<br>Education
**Brown 2021**	USA	Cohort	Community clinic and homeless shelters[F]	Medicine, Nursing, Pharmacy	n=1733<br>Marginalised	Vaccination
**Burgess 2022**	Australia	Qualitative	Hospital[*]	Medicine, Physiotherapy, Occupational Therapy, Speech Pathology, Pharmacy	n=11<br>Parkinson's Disease	Assessment
**Busen 2014**	USA	Mixed methods	Community clinic or centre and transitional living facility[F]	Nursing, Dentistry, Nutrition	n=12<br>Previously incarcerated women	Education
**Dacey 2010**	USA	Mixed methods	Assisted living facility[*]	Medicine, Nursing, Pharmacy, Psychology	n= not stated<br>Nursing home residents	Assessment<br>Therapy, group<br>Education

**Danhausen 2015**	USA	Descriptive; Retrospective chart review	Community clinic or centre[F]	Medicine, Midwifery	n=152<br>Uninsured vulnerable pregnant women	Assessment<br>Therapy, individual<br>Education
**Felder-Heim 2020**	USA	Mixed methods	Community clinic or centre[F]	Medicine, Physiotherapy, Dentistry, Pharmacy, Psychology, Care Coordination	n=105<br>Uninsured patients with diabetes or hypertension	Assessment<br>Therapy, individual
**Fröberg 2018**	Sweden	Mixed methods	Community clinic or centre[P]	Medicine, Nursing, Physiotherapy, Occupational Therapy, Psychology	n=938<br>Community residents	Assessment<br>Therapy, individual
**Fung 2022**	Hong Kong	Mixed methods	Telehealth[*]	Medicine, Nursing, Nutrition, Pharmacy, Public Health, Biomedical Sciences	n=25<br>Older adults	Assessment<br>Education
**Garavelis 2023**	Australia	Qualitative	Community clinic or centre[*]	Occupational Therapy, Psychology	n=10<br>Children and adolescents with suspected prenatal alcohol exposure	Assessment<br>Report writing<br>Client feedback session<br>Administrative tasks
**Gortney 2018**	USA	Descriptive	Church[F]	Medicine, Social Work, Pharmacy	n=233<br>Homeless people	Assessment<br>Therapy, individual<br>Education<br>Referrals<br>Medication review<br>Welfare
**Henderson-Kalb 2022**	USA	Qualitative	Community centre and telehealth[F]	Medicine, Nutrition, Occupational Therapy, Physiotherapy, Social Work, Speech Pathology	n=77<br>Underserved over 65 living in metropolitan area	Assessment<br>Recommendations and referrals
**Howell 2021**	USA	Mixed methods	Independent living housing community[*]	Dietetics, Nutrition, Kinesiology Public Health, Health Sciences	n=14<br>Older adults in housing community	Therapy, group<br>Education
**Hu 2016**	Canada	Descriptive; Retrospective chart review	52 Community clinics or centres[F]	Medicine, Nursing, Physiotherapy, Social Work, Pharmacy	n=268<br>Underserved with acute care issues	Assessment<br>Therapy, individual<br>Referrals<br>Medication review
**Janson 2009**	USA	non-RCT	Hospital[F]	Medicine, Nursing, Pharmacy	n=221<br>Diabetes	Assessment<br>Therapy, individual<br>Education

**Johnston 2019**	South Africa	Descriptive; Retrospective chart review	Church[F]	Medicine, Pharmacy	n=178<br>Inner city homeless people	Assessment<br>Referrals<br>Medication review
**Johnston 2020**	South Africa	Qualitative	Church[F]	Medicine, Pharmacy	n=18<br>Homeless people	Assessment<br>Therapy, individual<br>Referrals<br>Medication review<br>Welfare
**Kahkoska 2018**	USA	Descriptive; Cohort	Community clinic or centre[F]	Medicine, Nursing, Physician Assistant, Pharmacy	n=29<br>Uninsured with type 2 diabetes and HbA1c > 6.5%	Assessment<br>Therapy, individual<br>Education
**Kent 2013**	Australia	Descriptive	Community clinic or centre[*]	Medicine, Nursing, Physiotherapy, Occupational Therapy, Dietetics, Nutrition, Social Work	n=25<br>Over 70s	Assessment<br>Referrals
**Kent 2016**	Australia	Descriptive; Cohort	Community clinic or centre[*]	Nursing, Physiotherapy, Occupational Therapy, Speech Pathology, Dietetics, Social Work, Psychology, Podiatry	n=98<br>Recently discharged medical inpatients	Assessment<br>Referrals
**Krout 2010**	USA	Descriptive	Nursing home and housing community[*]	Physiotherapy, Occupational Therapy, Speech Pathology, Recreational Therapy, Audiology	n=357<br>Over 60s residents post stroke	Therapy, individual
**Lawrence 2015**	USA	Retrospective chart review; non-RCT	Community clinic or centre[F]	Medicine, Nursing	n=196<br>Local urban underserved population	Assessment<br>Therapy, individual<br>Education<br>Referrals<br>Clinic administrative tasks
**Leung 2012**	USA	Mixed methods	Community clinic or centre and telehealth[F]	Medicine, Nursing, Pharmacy	n=25<br>Uninsured, hypertensive patients	Education<br>Medication<br>Health coaching
**Liang En 2011**	Singapore	Mixed methods	Community clinic or centre, and home visit[F]	Medicine, Nursing	n=355<br>Low-income people living in public housing	Assessment<br>Education<br>Referrals<br>Medication review

**Meek 2013**	Australia	Mixed methods	Hospital[F]	Medicine, Nursing	n=734<br>Emergency department patients	Assessment<br>Therapy, individual<br>Education<br>Referrals
**Meuser 2022**	Australia	Qualitative	Telehealth[*]	Occupational Therapy, Social Work, Osteopathic Medicine	n=approx. 250<br>Community dwelling older adults	Therapy, group<br>Education<br>Social group activities
**Ng 2020**	Singapore	Descriptive; pre-post test	Home visit[*]	Medicine, Nursing, Physiotherapy, Occupational Therapy, Social Work, Pharmacy	n=116<br>Older adults with frequent hospital admissions	Assessment<br>Education<br>Medication review<br>Social group activities<br>Coordination of services
**Ouyang 2013**	USA	non-RCT	Community clinic or centre, and university clinic[M]	Medicine, Nursing, Pharmacy	n=138<br>New patients with hepatitis B virus	Education<br>Vaccination<br>Phlebotomy
**Palma 2020**	USA	Descriptive	9 Mobile clinics (truck/bus)[F]	Medicine, Nursing, Physician Assistant, Physiotherapy, Dentistry, Social Work, Pharmacy, Public Health	n=1305<br>Underserved people	Assessment<br>Therapy, individual<br>Education<br>Referrals<br>Vaccination
**Peluso 2014**	USA	Descriptive; Retrospective chart review	Community clinic or centre [F]	Medicine, Nursing, Physician Assistant	n=39<br>Foreign-born people with tuberculosis	Assessment<br>Education<br>Referrals<br>Welfare
**Reumerman 2021**	The Netherlands	Mixed methods	Hospital[*]	Medicine, Nursing, Physician Assistant, Pharmacy	n=32<br>Geriatric outpatients	Medication review
**Reumerman 2022**	The Netherlands	RCT	Hospital[*]	Medicine, Nursing, Pharmacy	n=216<br>Geriatric patients	Standard care + ISP (intervention group) vs standard care (control group)<br>Medication review
**Rock 2014**	USA	RCT	Home visit[F]	Medicine, Nursing, Social Work, Law	n=330<br>Underserved households	Student team (intervention) vs clinicians (control group)<br>Assessment<br>Education<br>Referrals

**Rowe 2021**	USA	Descriptive; Retrospective chart review	3 Community clinics or centres[F]	Nursing, Social Work, Pharmacy	n=50<br>Uncontrolled type 2 diabetes and/or a concomitant psychosocial condition	Assessment<br>Therapy, individual<br>Education<br>Referrals<br>Medication review
**Sargison 2021**	Australia	Qualitative	Pre-schools[F]	Occupational Therapy, Speech Pathology	n=9 (parents/ carers)<br>Aboriginal and Torres Strait Islander children	Assessment<br>Therapy, individual and group<br>Education
**Sarovich 2022**	Australia	Qualitative	Home visits and community sites[F]	Occupational Therapy, Physiotherapy, Speech Pathology, Dietetics, Social Work	n=6<br>Aboriginal and Torres Strait Islander people	Assessment<br>Therapy, individual and group<br>Referrals
**Sealey 2017**	Australia	Mixed methods	University clinic[P]	Medicine, Nursing, Physiotherapy, Exercise Physiology, Speech Pathology, Dentistry, Pharmacy, Psychology	n=14<br>Men aged 35 to 65, with a self-measured waist girth> 94 cm	Assessment<br>Therapy, group<br>Education
**Seymour 2010**	USA	Mixed methods	Home visits[*]	Nursing, Physiotherapy, Exercise Physiology	n= not stated<br>Community dwelling at-risk elders	Assessment<br>Education
**Shekar 2020**	USA	Descriptive	Detention centre[F]	Medicine, Nursing, Physician Assistant, Social Work	n=253<br>Youths in detention	Therapy, group<br>Education
**Sheu 2010**	USA	Descriptive	Community clinic or centre[M]	Medicine, Nursing, Dentistry, Pharmacy	n=804<br>Asian/Pacific Islander population at risk of Hepatitis B infection	Assessment<br>Education<br>Referrals<br>Vaccination<br>Clinic administrative tasks
**Sultan 2022**	The Netherlands	RCT	University hospital[*]	Medicine, Nursing, Physician Assistant, Pharmacy	n=216<br>Over 70s	Standard care + student team (intervention) vs standard care (control)<br>Medication review
**Virtue 2018**	USA	Longitudinal non-RCT	University clinic[*]	Dentistry, Pharmacy	n=50<br>Current smokers presenting for non-emergency dental care	Interprofessional team (intervention) vs standard care (control)<br>Assessment<br>Therapy, individual

						Education<br>Referrals<br>Medication review
**Walker 2022**	Australia	Qualitative	Telehealth*	Nursing, Physiotherapy, Exercise Physiology, Dietetics, Social Work, Psychology	n=14<br>Adults with low to rising risk of chronic disease	Assessment<br>Therapy, individual and group<br>Referrals<br>Social group activities

F = free clinic, P= low cost paid services, M= mixed free and low cost, PH = patient public health package, * = payment method not stated, RCT = randomised controlled trial

Article title: Impact of interprofessional student led health clinics for patients, students and educators: a scoping review
Journal name: Advances in Health Sciences Education
Author names: Janine Prestes Vargas, Moira Smith, Lucy Chipchase, Meg E. Morris
Affiliation of corresponding author: Victorian Rehabilitation Centre, Glen Waverley, and ARCH and CERI La Trobe University
Email of corresponding author: m.morris@latrobe.edu.au
