# Supplementary material for: Impact of interprofessional student led health clinics for patients, students and educators: a scoping review
Source: Adv Health Sci Educ Theory Pract. 2024 Jun 6;30(1):321–45. doi: 10.1007/s10459-024-10342-2 (PMC11925975; doi:10.1007/s10459-024-10342-2)
Supplement: Supplementary file 3 — Supplementary Material 3 [file 10459_2024_10342_MOESM3_ESM.pdf]

SUPPLEMENTARY MATERIAL 3: QUALITY ASSESSMENT OF INCLUDED STUDIES

	QUALITATIVE					QUANTITATIVE RCT					QUANTITATIVE NON-RCT					QUANTITATIVE DESCRIPTIVE					MIXED METHODS					
	Appropriate approach	Adequate methods	Findings derived from data	Interpretation of results	Coherence	Randomization	Groups comparable	Complete outcome data	Blinding	Adherence to group	Target population	Appropriate measures	Complete outcome data	Confounders accounted for	Appropriate intervention	Appropriate sampling	Target population	Appropriate measures	Low bias risk	Statistical analysis	Methods address research question	Integrated components	Adequate interpretation	Inconsistencies addressed	Adherence to quality criteria	**Total**
Asanad et al 2018																1	1	1	0	1						4/5
Beckman et al 2022																					1	1	1	1	0	4/5
Bird et al 2022	1	1	1	1	1																					5/5
Brown et al 2015											0	1	0	1	1											3/5
Brown et al 2021																1	1	1	1	1						5/5
Burgess & Roberts 2022	1	1	1	1	1																					5/5
Busen 2014																					1	0	0	0	0	1/5
Dacey et al 2010																					1	1	1	1	0	4/5
Danhausen et al 2015																1	1	0	0	1						3/5
Felder-Heim & Mader 2020																					1	1	1	1	1	5/5
Froberg et al 2018																					1	1	1	1	1	5/5
Fung et al 2022																					1	1	1	1	1	5/5
Garavelis et al 2023	1	1	1	1	1																					5/5
Gortney et al 2018																1	1	1	1	1						5/5

	QUALITATIVE					QUANTITATIVE RCT					QUANTITATIVE NON-RCT					QUANTITATIVE DESCRIPTIVE					MIXED METHODS					
	Appropriate approach	Adequate methods	Findings derived from data	Interpretation of results	Coherence	Randomization	Groups comparable	Complete outcome data	Blinding	Adherence to group	Target population	Appropriate measures	Complete outcome data	Confounders accounted for	Appropriate intervention	Appropriate sampling	Target population	Appropriate measures	Low bias risk	Statistical analysis	Methods address research question	Integrated components	Adequate interpretation	Inconsistencies addressed	Adherence to quality criteria	**Total**
Henderson-Kalb et al 2022	0	0	0	0	0																					0/5
Howell et al 2021																					1	1	1	1	1	5/5
Hu & Leung 2016																1	0	1	1	1						4/5
Janson et al 2009											1	1	1	1	1											5/5
Johnston et al 2019																1	1	1	1	1						5/5
Johnston et al 2020	1	1	1	1	1																					5/5
Kahkoska et al 2018																1	1	1	1	0						4/5
Kent & Keating 2013																1	1	1	0	1						4/5
Kent et al 2016																1	1	1	0	1						4/5
Krout et al 2010																1	1	0	0	0						2/5
Lawrence et al 2015											1	1	1	1	1											5/5
Leung et al 2012																					1	1	1	1	0	4/5
Liang En et al 2011																					1	1	1	1	1	5/5
Meek et al 2013																					1	1	1	1	0	4/5
Meuser et al 2022	0	0	1	1	0																					2/5

	QUALITATIVE					QUANTITATIVE RCT					QUANTITATIVE NON-RCT					QUANTITATIVE DESCRIPTIVE					MIXED METHODS					
	Appropriate approach	Adequate methods	Findings derived from data	Interpretation of results	Coherence	Randomization	Groups comparable	Complete outcome data	Blinding	Adherence to group	Target population	Appropriate measures	Complete outcome data	Confounders accounted for	Appropriate intervention	Appropriate sampling	Target population	Appropriate measures	Low bias risk	Statistical analysis	Methods address research question	Integrated components	Adequate interpretation	Inconsistencies addressed	Adherence to quality criteria	**Total**
Ng et al 2020																1	1	1	0	1						4/5
Ouyang et al 2013											1	1	1	0	1											4/5
Palma et al 2020																1	1	1	0	0						3/5
Peluso et al 2014																1	1	1	0	1						4/5
Reumerman et al 2021																					1	0	1	1	0	3/5
Reumerman et al 2022						1	1	1	1	1																5/5
Rock et al 2014						1	0	1	0	0																2/5
Rowe et al 2021																1	1	1	1	1						5/5
Sargison et al 2021	1	1	1	1	1																					5/5
Sarovich et al 2022	1	1	1	1	1																					5/5
Sealey et al 2017																					1	1	1	1	1	5/5
Seymour & Cannon 2010																					1	0	1	1	0	3/5
Shekar et al 2020																1	1	1	1	1						5/5
Sheu et al 2010																0	1	0	0	0						1/5
Sultan et al 2022						1	1	1	0	1																4/5

	QUALITATIVE					QUANTITATIVE RCT					QUANTITATIVE NON-RCT					QUANTITATIVE DESCRIPTIVE					MIXED METHODS					
	Appropriate approach	Adequate methods	Findings derived from data	Interpretation of results	Coherence	Randomization	Groups comparable	Complete outcome data	Blinding	Adherence to group	Target population	Appropriate measures	Complete outcome data	Confounders accounted for	Appropriate intervention	Appropriate sampling	Target population	Appropriate measures	Low bias risk	Statistical analysis	Methods address research question	Integrated components	Adequate interpretation	Inconsistencies addressed	Adherence to quality criteria	**Total**
Virtue et al 2018											1	1	0	1	1											4/5
Walker et al 2022	1	1	1	1	1																					5/5

1, criterion met; 0, criteria not met or unable to determine; RCT, randomised controlled trials.

Article title: Impact of interprofessional student led health clinics for patients, students and educators: a scoping review
Journal name: Advances in Health Sciences Education
Author names: Janine Prestes Vargas, Moira Smith, Lucy Chipchase, Meg E. Morris
Affiliation of corresponding author: Victorian Rehabilitation Centre, Glen Waverley, and ARCH and CERI La Trobe University
Email of corresponding author: m.morris@latrobe.edu.au
