# Supplementary material for: Impact of interprofessional student led health clinics for patients, students and educators: a scoping review
Source: Adv Health Sci Educ Theory Pract. 2024 Jun 6;30(1):321–45. doi: 10.1007/s10459-024-10342-2 (PMC11925975; doi:10.1007/s10459-024-10342-2)
Supplement: Supplementary file 4 — Supplementary Material 4 [file 10459_2024_10342_MOESM4_ESM.pdf]

SUPPLEMENTARY MATERIAL 4: PATIENT OUTCOMES

Study ID	Patient population	Patient outcome measures	Satisfaction	Experience/ perceptions	Health screening	Number of sessions attended	Demographics	Other
**Asanad 2018**	Homeless	Client satisfaction questionnaire	Most very satisfied, mean 3.8/5, SD 0.47. Highest satisfaction with pleasant and trustworthy staff, mean 3.9, SD 0.38. Satisfaction with cleanliness mean 3.7/5, as well as safety, medications, and hygiene kits. Prompt service 3.5, SD 0.69.	81.3% felt improved access to healthcare resources. 86.9% preferred this clinic over another free clinic. 89.6% preferred this clinic over emergency or non-emergency facility. 62.2% would recommend this clinic to others.	Not stated	Not stated	84.3% male, 25.8% first-time clients, 63.1% received medical care solely from this clinic	Not stated
**Beckman 2022**	Over 50s	Surveys	83% strongly agreed to be satisfied with care at the clinic. 92% overall satisfied with care they received.	78% felt confident in the ability of the students to be involved in their care, 92% recognised aspects of teamwork between the students.	Not stated	Not stated	Age range 65-79, 82.6% female	Not stated
**Bird 2022**	Older Yolŋu (aboriginal community residents) and their families	Interviews	Not stated	Older Yolŋu valued the time students spent with them, the attention, and the relevance of their questions. They also valued students were always happy and pleasant. From family members: support received by students made their	Not stated	Not stated	Not stated	Not stated

Study ID	Patient population	Patient outcome measures	Satisfaction	Experience/ perceptions	Health screening	Number of sessions attended	Demographics	Other
				loved one feel happy, family members valued the time students spent with clients.				
**Brown 2015**	Low-income overweight and obese community residents	Weight loss, attendance, comparison of outcomes in professional led and student led programs	Not stated	Not stated	Weight loss in both groups, $F(1,44)=45.99$, $P<.001$.	Patients attended more sessions and were more likely to complete program in professional group Professional group attendance = 6.86 vs 4.36in student group	43 (93.0%) female	Not stated
**Brown 2021**	Marginalised	Housing status, employment status, insurance status, frequency of influenza vaccination, vaccination delivered	Not stated	Not stated	Not stated	Not stated	Mean age 40.8 (17.2), 844 (48.7%) female, 481 (27.8%) homeless or in temporary housing, 673 (38.8%) unemployed. Most 1,097 (63.3%) not insured in previous two	1733 patients received influenza vaccine

Study ID	Patient population	Patient outcome measures	Satisfaction	Experience/ perceptions	Health screening	Number of sessions attended	Demographics	Other
							years, 49.5% took annual vaccine, this was a first ever vaccine in 21.8%.	
**Burgess 2022**	Parkinson's Disease	Semi-structured focus groups	Not stated	Theme 1: Interprofessional aspect. Communicating with students helped to build knowledge of patients' own healthcare needs. Theme 2: Students helped reflection on experiencing changes in health. Theme 3: Improved knowledge and access to healthcare. Theme 4: Patients wanting to assist with student education. Theme 5: Social aspect. Clinic as an additional means to meet others with Parkinson's disease.	Not stated	Not stated	5 female, 6 male	Not stated

Study ID	Patient population	Patient outcome measures	Satisfaction	Experience/ perceptions	Health screening	Number of sessions attended	Demographics	Other
**Busen 2014**	Previously incarcerated women	Hope for Health Seminar Evaluation sheet	Highly satisfied	Points highly rated (5): topic being interesting, learning new information, the wish to share information with loved ones, adequate course material, presenters answering questions and appropriate organization. Program allowed residents to learn, education topics were beneficial, fun, 'informative' and simple to learn, participants were treated with respect, useful knowledge of self-management, health monitoring and medications.	Many received dental treatment. Several with diagnosis of hypertension. One resident diagnosed with breast cancer after being educated on self-assessment.	4 sessions	Mean age 46.5 (35-55) Non-violent offenders with history of substance abuse. Residents at Brigid's Hope lived onsite between 1-11 months.	Not stated
**Dacey 2010**	Nursing home residents	Program satisfaction survey	80% reported to enjoy the program and to have learned about their health condition	70% gained knowledge of monitoring own health. Residents sustained lifestyle changes at 4 months, positive strategies implemented.	Not stated	2	Not stated	Programs included presentations, dietary information, educational games and relaxation sessions
**Danhausen 2015**	Uninsured vulnerable	Retrospective chart review, services	Not stated	Patients improved knowledge of early pregnancy program.	Many medical conditions reported and	Not stated	56 (36.8%) nulliparous	Women received timely referrals for continuing

Study ID	Patient population	Patient outcome measures	Satisfaction	Experience/ perceptions	Health screening	Number of sessions attended	Demographics	Other
	pregnant women	provided by clinic between 2010-2013			treated: gestational diabetes, gestational hypertension, abnormal Papanicolaou test, asthma, chlamydia, skin conditions, fatigue, abnormal pain/cramping, etc. Gestational ages: 79 (51%) <14 weeks, 51 (33.6%) 14-27 weeks, 12 (7.9%) >28 weeks, 10 (6.6%) unknown.		41 (27%) 1 prior birth 22 (14.5%) 2 prior births. Many did not have identification documents Majority in first trimester. Most were Latina women.	prenatal care if screening results were concerning. Counselling and social supports provided. Women received help to acquire health insurance.
**Felder-Heim 2020**	Uninsured patients with diabetes or hypertension	Adequate BP control (<140/90 mmHg) at the last appointment, HbA1c screen, nephropathy screen, retinopathy screen. Study clinic (DAWN) was compared to 3 other similar safety-net clinics.	Not stated	Self-reported health was 'fair' or 'poor'. Most did not have regular access to healthcare.	DAWN clinic had similar diabetes process outcomes compared to the 3 other similar clinics. Worse rates of blood pressure control compared to other 3 clinics 33.3% vs 49.2% ($p<0.01$), 61.1% ($p<0.01$) and 58.9% ($p<0.01$). 77.3% received a BP measurement	Not stated	54% females, 88% 18-64 years old, 100% clients at DAWN did not have health insurance. More than 90% had no access to primary health care before attending the clinic.	Social barriers: food, transportation, finances, and legal status.

Study ID	Patient population	Patient outcome measures	Satisfaction	Experience/ perceptions	Health screening	Number of sessions attended	Demographics	Other
					at every visit, 73.3% were prescribed appropriate anti-hypertensive medication. Mean number of medications prescribed: 5.67			
**Froberg 2018**	Community residents	Client satisfaction questionnaire-8 (CSQ-8) and 2 questions: 1. 'What worked well at the student-run clinic?' 2. 'What could have been done differently and better?'	Patient were very satisfied with care received. Mean CSQ-8 scores:<br><18 years: 29.99<br>18-64 years: 30.04<br>>65 years: 30.59<br>Unspecified: 29.95 (p=0.01)	Patients felt understood, well cared for, less stressed, respected and listened to by students. Patients perceived students and supervisors as knowledgeable and professional. Patients enjoyed being invited to discuss own treatment, they felt 'seen'. Longer appointments, punctuality and organization were appreciated, although some felt sessions took too long.<br>Some patients perceived students to be insecure as caregivers.	Not stated	Not stated	Mean age 54 (5-94)<br>52% women	Not stated

Study ID	Patient population	Patient outcome measures	Satisfaction	Experience/ perceptions	Health screening	Number of sessions attended	Demographics	Other
**Fung 2022**	Older adults	Participants' feedback survey (post-test), COVID-19-related knowledge questionnaire (pre and post), Geriatric Depression Scale (GDS15) (pre-test)	Most participants were highly satisfied with the intervention. They enjoyed the frequent contact with young people. All felt program was flexible and convenient. 28% preferred F2F interventions due to more interaction and 16% wanted F2F seminars. 46% felt more positive after the program.	Participants perceived the duration (96%) and content difficulty (88%) of phone calls to be appropriate. 12% found questions were too easy. 96% found program 'useful' and improved their COVID19 knowledge. 92% felt 'cared for', 'hopeful' and 'happy'. 12% wished for more notice of phone calls to improve their planning. 72% felt there was nothing to improve upon.	9 (36%) participants were at risk of developing depression in the GDS-15	5	Mean age 71.7 (SD 4.8), 64% female, 56% secondary or above education, 60% had chronic diseases diagnosed, average phone call duration 23.7 min (14.6)	% change in scores of COVID-19-related knowledge questionnaire= Theme 1 medication safety: 76 to 95.2%; Theme 2 healthcare voucher scheme: 64 to 88.8%; Theme 3 COVID-19 myth-busting: 78 to 93.2%.
**Garavelis 2023**	Children and adolescents with suspected prenatal alcohol exposure	Semi-structured interviews via phone or face-to-face	Most satisfied to attend clinic due to feeling validated and informed, but challenges were reported in dealing with the diagnosis and finding treatment afterwards. Caregivers noted staff and students were friendly, polite, organised and good communicators. Caregivers reported children enjoyed the time with all involved, and	Caregivers felt reassured, listened to and valued by students. They reported gaining knowledge of child's condition and needs, child report provided information on acquiring public funding. Biological parents described feelings of guilt and shame were alleviated by the clinic and increased	Not stated	Not stated	9 female, all Caucasian, age range 9-15	Not stated

Study ID	Patient population	Patient outcome measures	Satisfaction	Experience/ perceptions	Health screening	Number of sessions attended	Demographics	Other
			parents valued family-centred care.	their confidence. A lack of orientation on how to implement recommendations, acquire support services, deal with difficulties at home and the family impact were noted by caregivers, who also wished intervention was offered.				
**Gortney 2018**	Homeless	Interprofessional Clinic Homeless Satisfaction with Student Engagement Survey	Highly satisfied. Patients had positive experiences engaging with students.	98.7% felt respected, 98.1% felt students demonstrated sensitivity, 96.4% felt comprehended. 90.3% strongly agreed they received good health or medication education. Most felt the assistance received by students was valuable.	Treatment delivered to those with chronic or skin conditions. Poor social determinants of health: housing, food, and access to health care.	Not stated	Mean age 54.27 (SD 11.3) 78% male 93.1% African American 4.6% White 2.3% other	Not stated
**Henderson -Kalb 2022**	Underserved over 65s living in metropolitan area	Feedback about experience via email	Most reported positive experiences, 'marvellous', 'nice experience'. Patients enjoyed speaking with a lot of people in the clinic.	Most felt comfortable, grateful, welcomed, and enjoyed spending time with the students. Patients reported to value the assessments and	Not stated	Not stated	Not stated	Not stated

Study ID	Patient population	Patient outcome measures	Satisfaction	Experience/ perceptions	Health screening	Number of sessions attended	Demographics	Other
				recommendations, and the wealth of information received.				
**Howell 2021**	Older adults in housing community	Focus groups, semi-structured interviews, participant satisfaction survey. Interprofessional student team vs usual care (medical residents)	All 7 respondents were satisfied or highly satisfied with student performance and quality of content. Most valuable aspect was having their voices heard while assisting students to design educational content, learning new exercises and nutritional tips, improved balance. Some valued the social and psychological benefits of the course, and one resident reported 'feeling loved'.	Patients felt able to use knowledge learned in daily life, valued the potential to improve their health, the impact on quality of life and the social contact. Too many different students rotating made it hard to get to know each and form relationships. Residents wished for a quieter room for the lectures. Some suggested education topics should be more tailored to the audience.	Not stated	Not stated	Not stated	Not stated
**Hu 2016**	Underserved with acute care issues	Wait times, total treatment time, total time per patient, reason for clinic visit, outcomes of visit	Not stated	Not stated	Not stated	Not stated	Not stated	Average time waited 21 min (0-73min). Average treatment time per patient was 69min (43-123min). Average total time spent in the clinic: 91min (75-153). 81 patients referred to ED. 52

Study ID	Patient population	Patient outcome measures	Satisfaction	Experience/ perceptions	Health screening	Number of sessions attended	Demographics	Other
								patients received prescriptions. Top 3 reasons to visit the clinic: physiotherapy, musculoskeletal issues, pain
**Janson 2009**	Diabetes	BP, foot exam HbA1c, LDL-C, MA, patient-centred goals, referrals	Not stated	Not stated	Intervention group more frequently received screening of HbA1c, LDL-C, MA, BP, smoking status and foot exams. LDL-C improved in both groups, no clinical status measures changed significantly for both groups at the end of the study. Poor attendance linked with higher BP and a tendency to not have ACE/ARB prescribed.	Intervention participants had 1.1 visit more than control participants. Intervention group had more planned medical appointments and fewer ED visits and hospital admissions.	Total sample mean age 64, 118 (53%) female, 68% insured by Medicaid or Medicare, 33% non-English speaking.	Not stated
**Johnston 2019**	Inner city homeless	BP, HR, medication dispensed, diagnosed conditions, referrals	Not stated	Not stated	BP ranged 88-214/50-116 mmHg. 16% with HT stage 1, 2 patients with hypertensive	81% visited the clinic once	Mean age 37.1 (18-73), 80% male.<br>76 smoked cigarettes, 80 consumed	Referrals given to 42%. 100 referred to a community health care clinic and public hospital. 28

Study ID	Patient population	Patient outcome measures	Satisfaction	Experience/ perceptions	Health screening	Number of sessions attended	Demographics	Other
					crisis. HR ranged 50-120bmp. Most common conditions: respiratory tract infections, digestive diseases and infectious and parasitic diseases. Medications: 38.8% prescribed analgesics, 13.8% antibiotics, 10.4% antihistamines and 8.3% vitamins. Five patients diagnosed with mental health conditions.		alcohol and 17 used recreational drugs. 74 chronic conditions documented, under half of those patients were on medication. 2 or more comorbidities in 11 patients. 31 (17.4%) knew being HIV+. 17 previously had TB. 3 had active TB under treatment.	referred to community health centre with suspected TB and HIV. 7 treated with syndromic treatment approach secondary to sexually transmitted diseases.
**Johnston 2020**	Homeless	Focus groups	Student clinic at the church felt like home to many. Services were appreciated, patients felt respected, encouraged and staff were compassionate, professional, and trustworthy. They valued longer time for consultations.	Homelessness affecting health: poor nutrition, poor hygiene, limited shelter, belongings are often stolen, common diseases reported were influenza, tuberculosis, sexually transmitted diseases and dental infections, drug/alcohol	Not stated	Not stated	15 men, 3 women, predominant language was English.	Not stated

Study ID	Patient population	Patient outcome measures	Satisfaction	Experience/ perceptions	Health screening	Number of sessions attended	Demographics	Other
				addiction. Homeless feel discriminated against in government clinics and not trusted with HIV meds. Homeless people wanted more frequent and varied services, including dentistry, social and psychological services.				
**Kahkoska 2018**	Uninsured with type 2 diabetes and an HbA1c > 6.5%	HbA1c, number of patients seen, wait times	Not stated	Not stated	Mean (SD) HbA1c at baseline: 9.7% ± 1.7% (83 ± 7 mmol/mol) and follow-up: 9.2% ± 1.8% (77 ± 8mmol/mol). Median HbA1c pre-test: 9.5% (80 mmol/mol) and post: 8.9% (74 mmol/mol). 6/8 patients showed decreased HbA1c after clinic. High between-person variability in response of glycaemic control to clinic.	Not stated	Not stated	SMA model accommodated 10-12 patients per clinic instead of 6-8 under traditional approach. SMA model decreased time waiting in isolation in waiting room as all patients are scheduled at the same time. SMA model increased time receiving education and patient-directed discussion.
**Kent 2013**	Over 70s	PEQ	Not stated	64% response rate. 94% reported feeling better prepared to	Urinary tract infection, pneumonia, and	1	Mean age 79 (SD 7.3), mean duration of	Most referrals written to address non-

Study ID	Patient population	Patient outcome measures	Satisfaction	Experience/ perceptions	Health screening	Number of sessions attended	Demographics	Other
				deal with own problems. 94% believed interprofessional care resulted in improved health. All patients indicated they were well informed of and consulted about important decisions.	gastroenteritis most frequently diagnosed during hospital admission.		previous hospital admission 6 days (SD 3.6), seen by students on average 17 (SD = 7) days post discharge.	urgent conditions, and were preventative in nature.
**Kent 2016**	Recently discharged medical inpatients	PEQ, demographics, referrals, hospital readmissions	Most patients felt reassured, understood, and had positive experiences from participating.	85% reported they improved knowledge of reducing health issues and 79% felt better prepared to deal with own health problems. 60 minutes of session were valued by patients.	Reasons for acute hospital admission: digestive and cardiovascular complaints. Duration of hospital stay 4 (1-17) days. Readmissions to hospital at 6 months: 33 patients (39%)	1	Mean age 76 (SD 7)	120 referrals written, most common for physio and podiatry. Health issues identified: rectal bleeding, depression, wound infection. Many student recommendations were declined by patients.
**Krout 2010**	Community residents post stroke	Satisfaction survey	All found the activities enjoyable. 98.7% pleased with program activities. Overall, a valuable experience on a personal level for 96.2%. 98.8% found the activity well organised.	91.5% thought the supervision was adequate, and all valued the adequate time spent with students. 94% found interactions with faculty were positive. 92% found the experience helped them to better	Not stated	Not stated	Not stated	Not stated

Study ID	Patient population	Patient outcome measures	Satisfaction	Experience/ perceptions	Health screening	Number of sessions attended	Demographics	Other
				understand students. 93.1% would continue working with students.				
**Lawrence 2015**	Local urban underserved population	Satisfaction survey	91% completed satisfaction surveys. 24 of 28 items given a rating of "good" to "great"	Highest satisfaction score was towards students answering questions. Low satisfaction with wait times in student run clinic. Most satisfied with cleanliness and privacy in both clinics. Greater levels of satisfaction were seen in non-student clinic in wait times, hours open, info privacy and likelihood to recommend to others.	Most common reasons for clinic visit: sexually transmitted infections (41%), work/school physical (33%).	Not stated	53% female<br>>80% African Americana<br>74% within ages 18-44	No significant differences between student and non-student clinic across quality of interactions with provider or cleanliness and comfort.
**Leung 2012**	Uninsured, hypertensive patients	Satisfaction, blood pressure, medication adherence, self-monitoring frequency, health behaviour, interviews, surveys	90% reported the intervention improved their self-efficacy (they were effective in improving hypertension), knowledge of healthier choices and felt more awareness and empowered to look after own health.	75% complied with self-assessment of blood pressure pre- and post-intervention. 92% felt they made progress in lifestyle goals: exercise, diet, weight loss, less alcohol intake.	76% had poorly controlled BP pre-test (M=141/89, SD=17.7). Daily pills: 1.5-12. 33 BP medications were prescribed. Significant improvements in systolic BP: pre 146.5 (18.68) and post 135.5 (18.34).	12 (48%) fully attended (2 visits + 6 phone calls).<br>88% completed half and 76% almost completed, 13 (52%) did not	60% male<br>52% Latino<br>24% Black<br>24% White<br>48% income between $0-9,999<br>52% some high school or graduates<br>All obese or overweight<br>84% non-smokers	Morisky Scale scores (medication adherence level at baseline): 4 to 8= medium adherence (low adherence<6)<br>Change to adherence level: Brief Medication Questionnaire: 92% of completers had a

Study ID	Patient population	Patient outcome measures	Satisfaction	Experience/ perceptions	Health screening	Number of sessions attended	Demographics	Other
						complete intervention		positive regimen at baseline. Post test, 25% improved medication adherence. 75% reported increased frequency of home BP monitoring.
**Liang En 2011**	Low-income community in public housing	Satisfaction, clinical outcomes, patient interviews	All participants extremely satisfied.	75% felt their health improved, 85% felt there was enough time to discuss their health concerns with the student.	Rates of chronic disease screening improved: hypertension screening from 36% to 99%; diabetes mellitus from 35% to 40% and dyslipidaemia from 26% to 30%, colorectal cancer from 6% to 16%. By 2010, BP control among 82 with known diagnosis of hypertension improved significantly from 42% to 79%. Treatment rates among those with hypertension increased from 63% to 93%. 49%	Not stated	35% >70yo, 54.4% female 55.5% Chinese 29% Malay 12.4% Indian 2.5% other 28.7% on financial aid 39.7% had diagnosis of hypertension, 21.4% diabetes 25.1% dyslipidaemia	Not stated

Study ID	Patient population	Patient outcome measures	Satisfaction	Experience/ perceptions	Health screening	Number of sessions attended	Demographics	Other
					achieved BP control 1 year later.			
**Meek 2013**	Emergency department patients	Australasian Triage Scale, waiting times, diagnostic group and disposition (discharge, ward admission, short stay unit admission), ED LOS, satisfaction survey. Student-led beds vs control beds.	34% response rate. Good to excellent score given by patients for student identification and education on their role (84%), treatment information (88%), clinical care received (91%) and satisfaction (93%).	Not stated	Not stated	Not stated	Median age 54<br>50% male<br>Diagnosis group:<br>medical 48%<br>surgical 23%<br>injury 21%<br>psychiatry 7.9%<br>Inpatient admission 51%<br>Short stay 30%<br>Discharged 19%	Median time to see a doctor or nurse was not statistically different between groups. ATS category 3 patients were attended to within target time by students (22% vs 14%, P=0.04). More patients in control beds admitted as an inpatient within 2h (57% vs 45%, P=0.03).
**Meuser 2022**	Community-dwelling older adults	Online feedback on the program	Not stated	Telecollaborative service-learning project mediated effects on social isolation. Participants enjoyed advancing their knowledge of technology with students. One person valued the 'positivity' and 'normalcy' associated with the program. Participants remarked the	Not stated	Most attended multiple sessions	Over 55s	Legacy Scholars Program: research registry for adults over 55, longitudinal study of healthy ageing. Prior to pandemic, scholars gathered every 2 months to discuss general topics. This group was targeted first for recruitment.

Study ID	Patient population	Patient outcome measures	Satisfaction	Experience/ perceptions	Health screening	Number of sessions attended	Demographics	Other
				program was of mutual benefit to them and students.				
**Ng 2020**	Older adults with frequent hospital admissions	Patient feedback survey pre and post, number of hospital admissions and emergency department visits 6 months before and after the program	95% enjoyed activities during home visits	80% felt happier and less lonely, 71.4% felt knowledge of own health problems improved, 68% felt more confident in self-care, 50% improved lifestyle, 96% reported students were respectful, 82.4% found easy to communicate with students, most thought the duration of visits was adequate but 9.8% thought it was too short and 2% too long.	Not stated	12	Mean age 73.5 (54-95), 54.7% male, Charlson Comorbidities Index 9.1 (SD 2.9)	Statistically significant decrease in hospital readmissions between 6 months pre and post program from 1 (0-5) to 0 (0-10), and emergency department 0 (0-10) to 0 (0-10) 6 months post.
**Ouyang 2013**	New patients with hepatitis B virus (HBV)	Knowledge survey at start and end of initial session and once again at 1-month follow-up before screening results	Not stated	Knowledge survey scores: time-point 1: 56.4% (SD=15.3%), time-point 2: 66.6 % (SD=15.1%), time-point 3: 68.3% (SD=15.2%). Statistically significant between 1 and 2 and 1 and 3 but not between 2 and 3.	Not stated	2	Mean age 56.4 (SD=17.3), 67.6% female, 56% primary language not English, 50% had completed school or less, 64.2% income <25K.	Time-point 1: 68% correct answers about risk factors for transmission of hepatitis B. Score improvement was correlated with higher education.

Study ID	Patient population	Patient outcome measures	Satisfaction	Experience/ perceptions	Health screening	Number of sessions attended	Demographics	Other
**Palma 2020**	Underserved	Satisfaction survey	Percentage of patients rating care as: Excellent 69.1%, Good 27.9%, Fair 2.9%, Poor 0%	89.8% thought the service completely met all needs and answered all questions, others thought this happened to some extent (7.2%) or not at all (2.8%).	Not stated	98.8% attended 1 session only, 1.2% had a follow-up visit	54.4% over 18, 52.1% first time using the clinic 5.7% used clinic monthly 37.6% used clinic a few times a year 4.3% every few years	Clinic was the only source of healthcare for 44% of patients surveyed
**Peluso 2014**	Underserved high-risk, foreign-born with latent tuberculosis infection (LTBI)	Therapy adherence, side effects, and outcome data in three program outcomes: 'completion', 'managed discontinuation' , and 'lost to follow-up'	Not stated	Barriers to taking tuberculosis medication: forgetting to take, side effects, desire to drink alcohol, household duties. 41% reported no barriers.	67% completed 9 months of tuberculosis therapy, 21% discontinued due to side effects, 12% were lost to follow-up. Side effects: Fatigue 31%, Jaundice 13%, Nausea/ vomiting 21%, Abdominal pain 38%, Paraesthesia 26%, Other 56%.	Median 9 (range 1-13)	Median age 34 (21-54), 56% male, 92% Spanish-speaking immigrants. All ineligible to receive health insurance due to immigration status. 26% completed secondary school. Time since last medical appointment: < 1 year: 31% 1-5 years: 18% > 5 years: 18% Don't remember: 33%	Mean self-reported medication adherence: 29/30 pills per month (96%). Median number of treatment days: 273 among completers, 95 among those who discontinued, and 63 among those lost to follow-up. 1 death related to complication of substance toxicity. Those who discontinued had more encounters per month than completers, due to more side effects.

Study ID	Patient population	Patient outcome measures	Satisfaction	Experience/ perceptions	Health screening	Number of sessions attended	Demographics	Other
**Reumerman 2021**	Geriatric outpatients attending a memory clinic	Review of electronic patient healthcare records, medication lists, type of advice given and implemented, medications reviewed	Not stated	Not stated	Dementia frequently diagnosed (46.9%), followed by mild cognitive impairment (31.2%). Median 6 (3-8) medications per patient. Advice given (and implemented): stop medication 23%, start medication 23%, switch medication 9%, adjust dosing 3%, monitoring advice 8%. Most drugs involved in advice were cardiovascular 33%, osteoporosis prevention 24% and gastrointestinal 11%.	1	Median age 80, 59.4% male	No one had medications reviewed in past 12 months. A total of 188 medications were reviewed and 95 changes were recommended, which 80 of those were accepted in multidisciplinary meeting and 68 directly implemented. IP student teams detected 14 potential medication-related issues. Medical problems were not picked up by IP team for 2 patients with rigidity and orthostatic hypotension.

Study ID	Patient population	Patient outcome measures	Satisfaction	Experience/ perceptions	Health screening	Number of sessions attended	Demographics	Other
**Reumerman 2022**	Geriatric patients suspected of cognitive decline	Adverse drug reactions (ADR) reported in the medical record, severity and advice to treat. Patient phone interview at 3-months (conducted by a clinical pharmacologist)<br><br>Groups Control: Standard care vs Experimental: standard care + ISP team	Not stated	Not stated	ADRs detected Mild:38 Moderately severe: 20. More ADRs detected in the experimental group (n = 48) vs control (n = 10; p < 0.001).<br>Of those 48, 4 detected by a doctor but missed by the IP team, 5 detected by both groups, 44 detected by experimental groups but missed by doctor.	2	Median comorbidity index of 5	67-80% of ADRs were deemed preventable.
**Rock 2014**	Underserved households	Surveys to check preventive measures adherence and use of emergency department. 'Behavioral Risk Factor Surveillance System', 'Pregnancy Risk Assessment Monitoring System', 'National Health	Not stated	Not stated	Before household visits, student-visited households used all the preventive interventions less, except for sigmoidoscopy or colonoscopy.	Not stated	In both groups, half had less than high school. Most retired or not employed, on <30K income. Half with good/excellent health. Most had no health insurance. Most common conditions: hypertension, diabetes, anxiety,	Intervention households were more likely to adhere to most of the preventive health measures. Emergency department visits were less frequent in both groups at follow-up, with greater tendency toward decreased use in the intervention group (OR 0.4, 95% CI 0.09 -

Study ID	Patient population	Patient outcome measures	Satisfaction	Experience/ perceptions	Health screening	Number of sessions attended	Demographics	Other
		and Nutrition Examination Survey'					depression, obesity and asthma.	1.81). At 1 year, intervention group had more annual physical examinations, cervical screenings, mammograms, and were 16% more compliance with BP monitoring.
**Rowe 2021**	Uncontrolled type 2 diabetes and/or a psychosocial disorder	Glycosylated haemoglobin A1c (HbA1c) levels, BP, cholesterol, emergency department visits 6 months pre- and post-participation, PHQ-2, PHQ-9	Not stated	Not stated	Mean systolic BP decreased significantly, from 129.3 (SD 15) to 124.8 (SD 13.1). 38 patients with diabetes decreased haemoglobin A1c from 11.1% (SD 2.2) to 8.9% (SD 2.1) by 6 months post ($p<.001$), this was equivalent to 35% reduction in long-term complications.	Not stated	Not stated	Emergency department visits reduced by 75% between 6 months pre and post. Statistically significant decreased PHQ-2 and PHQ-9 scores, potentially indicating decreased impact on mental health..
**Sargison 2021**	Aboriginal and Torres Strait Islander children	Interview or focus group	Not stated	Parents reported positive experiences and thought students were helpful and appropriate to the community. Parents felt reassured, saw	Not stated	Not stated	Indigenous children between 3-5 years old	Not stated

Study ID	Patient population	Patient outcome measures	Satisfaction	Experience/ perceptions	Health screening	Number of sessions attended	Demographics	Other
				the program as effective, and helpful in improving child's confidence in selfcare tasks and communication skills. Parents valued students' assistance, care, and attention with their child. Student ongoing communication with parents was valued and reported to increase parent engagement, however one parent perceived lack of communication and wished for more opportunities to meet and greet. Observation days and discussions with parents, early childhood educators and supervisors prior to clinic was valued and helped to deliver a client-centred service.				
**Sarovich 2022**	Older Aboriginal and Torres Strait	Interviews	Not stated	Four themes: 'Connection to past', 'Connection to people', 'Connection to future', 'Sense of	Not stated	Not stated	Aboriginal: 4, Aboriginal and Torres Strait Islander: 2, age range 62-67	Not stated

Study ID	Patient population	Patient outcome measures	Satisfaction	Experience/ perceptions	Health screening	Number of sessions attended	Demographics	Other
	Islander people			achievement and fun'. Most valued a connection with past experiences as well as using relevant familiar objects in therapy. Some activities were linked to independence and self-efficacy. Program was an opportunity for social contact in a fun and relaxed environment. Participants felt their cultural identity was strengthened by the program. They valued mutual learning and sharing knowledge with university students. Participants enjoyed activities and felt a sense of achievement and pride. Yarning facilitated rapport and respect between Elders and students.			years, several co-morbidities. Female: 5Male: 1	
**Sealey 2017**	Men 35-65 years old + self-measured waist girth> 94 cm	Pedometer readings, height, mass, waist girth, resting BP, IPAQ, SF12,	All thought education sessions were extremely or very useful. Patients reported students were knowledgeable and professional, education	One participant mentioned feeling more energetic and less breathless, better sleep, more relaxed. Participants	Non-statistically significant changes: BP, waist girth, mass, and BMI. Patients	Average 11 (range 9-12)	Mean age 50 (SD 8, range 35-64)	Group average daily step count 6,600 steps, range 1,500-8,700, numbers of steps remained

Study ID	Patient population	Patient outcome measures	Satisfaction	Experience/ perceptions	Health screening	Number of sessions attended	Demographics	Other
		health knowledge questionnaire created by the students	sessions were informative and relaxed, positive comments towards self-care education and health screening.<br>All would recommend to others. All felt enhanced health knowledge.	felt motivated to participate because they were overweight, had health conditions and liked a group setting. They enjoyed the organisation, tailored exercises, and supervised sessions.	improved lifestyle related to exercise, diet, dental hygiene, and medications. Statistical significance in physical and mental function (SF12) and recall of weekday sitting total (IPAQ).			the same during intervention.
**Seymour 2010**	Community dwelling at-risk elders	Health Promotion Inventories (HPI) included: Physical Performance Test, Lubben Social Network Scale, Geriatric Disability Scale, Mini-nutritional Assessment, BMI.	Not stated	Improved knowledge of how to prevent hospital readmissions. Socialization aspect was the best part of the visit.	Number of hospital readmissions at six months post was related to chronic obstructive pulmonary disease and depression. At 12 months, readmissions were related to cardiovascular disease. There was no significant decrease in readmissions post program but there was a slight correlation between comorbidities and	12	Not stated	Significantly improved scores in Physical Performance Test and Geriatric Depression Scale compared to a similar group who did not to participate in the program.<br>Positive trend in favour of the intervention group in Lubben Social Network Scale and Mini Nutritional Assessment.

Study ID	Patient population	Patient outcome measures	Satisfaction	Experience/ perceptions	Health screening	Number of sessions attended	Demographics	Other
					readmission at 6 months and slightly higher likelihood at 12 months.			
**Shekar 2020**	Youths in detention	Three self-administered surveys: baseline and end of day 2 and day 3. Subscale 'Say No' of the 'Sexual Self-Efficacy Scale', 'Violence Related Behaviors and Beliefs Insert'	Not stated	Not stated	Not stated	3 x 2-hour education sessions over 3 days	Age 12-19, 30/253 female. Subset of 17-19 years old improved in each category. Adolescents 12-14 years old did not improve. Females showed improved self-efficacy scores only.	Significant improvement in sexual self-efficacy in males only, from 74.5% to 81.2%, knowledge of importance of consent, from 80.2% to 85.5%, and willingness to intervene against sexual violence, from 73.6% to 77.8%. Subset 12-14 years old: no change. Subset 15-16 years old: no improvement in willingness to intervene, which was potentially related to lower sexual and emotional maturity.

Study ID	Patient population	Patient outcome measures	Satisfaction	Experience/ perceptions	Health screening	Number of sessions attended	Demographics	Other
**Sheu 2010**	At-risk Asian/Pacific Islander population	Serologic testing and vaccine use at each clinic	Not stated	Not stated	HBV-susceptible patients were encouraged to get vaccinated. 90% of these completed or were on track to complete all vaccinations, which is higher than reported in literature. Serologic testing: 10% were HBV-infected, 44% were HBV-susceptible, 46% were HBV-immune.	Not stated	Mean age 49 (SD 16), 55% female. 87% first-generation Asian/Pacific Islanders immigrants from 14 countries. 63% limited English 46% uninsured	Not stated
**Sultan 2022**	Over 70s, suspected of cognitive decline	Number of STOPP-START items identified by standard care (resident) and by the ISP team vs a review panel at baseline, number of items recommended in letter to GP, number of items implemented at 6 weeks	Not stated	Not stated	ISP team recommended nine times more medications than residents (128 vs 14). 56% diagnosed with dementia during the clinic.	2	Mean age 79, median comorbidity index 5, median 5 medications used.	Standard care: 24% of STOPP/START items identified by a review panel were identified by residents vs 62% by resident + ISP team. Of these, 9% of recommendations by residents were implemented by GPs at 6 weeks vs 19% residents + ISP team.

Study ID	Patient population	Patient outcome measures	Satisfaction	Experience/ perceptions	Health screening	Number of sessions attended	Demographics	Other
**Virtue 2018**	Current smokers presenting for non-emergency dental care	Follow-up questionnaire at 4 weeks via telephone<br><br>IP student team vs standard care groups	Mean satisfaction 4.74 ± 0.69, range 1-5<br>82.6% extremely satisfied, 13% satisfied, 4.3% unsatisfied.<br>Most patients valued learning about quit smoking strategies. Most were interested in discussing smoking cessation with a dentist-pharmacist team	Patients in student teams felt more knowledge gained and had more intentions-to-action compared to standard care. Standard care group did not correlate knowledge gained with other variables. Student team patients had slightly higher intentions to quit and higher intentions to use medications.	Not stated	1 dental appt, 1 phone interview 4 weeks after	Mean age 48 (SD 13), 64% female, 66% Black/African American, 80% non-Hispanic Latino, 68% had health insurance. 68% of pts in the standard care had at least some college education vs 32% in IP student team.	Study completers smoked less cigarettes per day compared to drop outs (Mcompleters = 6.16, Mdrops= 9.85; p= .044). Majority had at least 1 quit attempt. 65% of IPC tried to quit smoking, compared to 53% control. One in intervention quit, two cut down. Two in control quit, four cut down. 82% of intervention contacted a provider for more help, compared to 40% in control. Control perceived higher knowledge at follow-up than after dental visit, whereas IPC felt the same knowledge at 4 weeks.

Study ID	Patient population	Patient outcome measures	Satisfaction	Experience/ perceptions	Health screening	Number of sessions attended	Demographics	Other
**Walker 2022**	Adults with low to rising risk of chronic disease	Electronic survey	Not stated	Benefits and disadvantages of telehealth reported: (+) continuity of service and reduced travel time, (-) not being able to use gym and pool facilities and not receiving hands-on services. Telehealth reduced client ability to socialise with others, and to receive timely technique correction and advice.	Not stated	Not stated	4/14 clients completed online survey. All were receiving face-to-face services prior to COVID19 and telehealth clinic.	Not stated

Key: ACE/ARB: angiotensin-converting enzyme/angiotensin receptor blocker, ADRs: adverse drug reactions, BMI: body mass index, BP: Blood pressure, ED: emergency department, F2F: face-to-face, GP: general practitioner, HBV: hepatitis B viral; HR: heart rate; HT: hypertension IP: interprofessional, IPAQ: International Physical Activity Questionnaire Short Form, ISP: interprofessional student-run medication review program, LOS: length of stay, PEQ: Patient Experience Questionnaire, PHQ: Patient Health Questionnaire, SD: standard deviation, SF12: Short Form 12 Health Survey, TB: tuberculosis

Article title: Impact of interprofessional student led health clinics for patients, students and educators: a scoping review
Journal name: Advances in Health Sciences Education
Author names: Janine Prestes Vargas, Moira Smith, Lucy Chipchase, Meg E. Morris
Affiliation of corresponding author: Victorian Rehabilitation Centre, Glen Waverley, and ARCH and CERI La Trobe University
Email of corresponding author: m.morris@latrobe.edu.au
