# Supplementary material for: Impact of interprofessional student led health clinics for patients, students and educators: a scoping review
Source: Adv Health Sci Educ Theory Pract. 2024 Jun 6;30(1):321–45. doi: 10.1007/s10459-024-10342-2 (PMC11925975; doi:10.1007/s10459-024-10342-2)
Supplement: Supplementary file 5 — Supplementary Material 5 [file 10459_2024_10342_MOESM5_ESM.pdf]

SUPPLEMENTARY MATERIAL 5: STUDENT OUTCOMES

Study ID	Student disciplines	Number of students	Student admission to the clinic	Pre-clinic orientation or training	Student outcome measures	Satisfaction	Experience/perceptions	Time spent in the clinic	Other results
**Asanad 2018**	Medicine, Social Work, Public Health	Not stated	Mixed, placement and volunteer	'Formal training' completed by all members of the team	Not stated	Not stated	Not stated	Not stated	Not stated
**Beckman 2022**	Nursing, Physiotherapy, Occupational Therapy, Exercise Physiology, Dietetics, Social Work, Psychology, Counselling	31	Placement, elective	Not stated	Surveys and focus groups	Not stated	60% of students neutral in reporting confidence working interprofessionally prior to placement. Following placement, 65% felt improved interprofessional skills and teamwork, 73% improved knowledge of the role of other health disciplines which in turn improved confidence to work in a team. They improved present and potentially the future clinical practice. Students valued a holistic approach and timely feedback from educators. Student lacked clarity about the role of other professions, had differing communication styles and terminology and suggested more orientation to new	<2 weeks (3.2%), 4 weeks (6.5%), 5 weeks (29%), 6 weeks or more (61.2%)	Not stated

Study ID	Student disciplines	Number of students	Student admission to the clinic	Pre-clinic orientation or training	Student outcome measures	Satisfaction	Experience/perceptions	Time spent in the clinic	Other results
							students about their role in the clinic.		
**Bird 2022**	Occupational Therapy, Speech Pathology	4	Placement, elective	Three weeks of orientation to aboriginal community and culture, meeting and observing clients prior to clinic.	Interviews	Working within a remote aboriginal community was highly valued	Students learned how to use their skills to assist older people, learned about the aboriginal community and culture, communication skills and a holistic approach. Communicating with visiting services and other students was difficult at first but gains in knowledge of the other professions improved their communication skills. Preparation and orientation were essential to meet and observe support workers and clients prior to service delivery, this allowed rapport building and a true immersion in the aboriginal culture.	8 weeks	Not stated

Study ID	Student disciplines	Number of students	Student admission to the clinic	Pre-clinic orientation or training	Student outcome measures	Satisfaction	Experience/perceptions	Time spent in the clinic	Other results
**Brown 2015**	Medicine, Nursing, Health Sciences	35	Volunteer	Video recordings created with basic info on obesity. Weekly group meetings with advisors for the duration of the clinic to discuss experiences in delivering class and review upcoming class.	Not stated	Not stated	Not stated	Not stated	Not stated
**Brown 2021**	Medicine, Nursing, Pharmacy	Not stated	Volunteer	Orientation on vaccine handling and application by a registered nurse.	Not stated	Not stated	Not stated	Not stated	Not stated
**Burgess 2022**	Medicine, Physiotherapy, Occupational Therapy, Speech Pathology, Pharmacy	30-40	Not stated	30-minute orientation by a staff member, provided with assessment form to as guidance	Not stated	Not stated	Not stated	Not stated	Not stated
**Busen 2014**	Nursing, Dentistry, Nutrition	Not stated	Placement, elective and required	Seminars focused on team building; communication skills delivered during the first 4 weeks of the semester	Likert scale and open-ended evaluation questions	'Rigorous', 'valuable' and 'thought provoking' course. Rewarding experience.	'Fascinating' topic. Students had limited knowledge of scope of practice of other disciplines and reported learning a lot. Students improved perceptions related to altruism and	4 x 2h-session over 12 weeks	Students reported a beneficial relationship with supervisors and community.

Study ID	Student disciplines	Number of students	Student admission to the clinic	Pre-clinic orientation or training	Student outcome measures	Satisfaction	Experience/perceptions	Time spent in the clinic	Other results
							valued the social awareness.		
**Dacey 2010**	Medicine, Nursing, Pharmacy, Psychology	10	Volunteer	Lecture, role-play, case studies, peer editing, presentations, weekly didactic content on IP topics	RIPLS	Not stated	Students improved patient-centred awareness after the course. Students reported enhanced communication skills and respect towards other professions, and increased confidence to work in a team. They improved attitudes towards older adults. A student reported the importance of being adaptable.	1 semester	Not stated
**Danhausen 2015**	Medicine, Midwifery	~200	Volunteer	Orientation to clinic layout, resources and care procedures. Clinical flow sheet guided history and physical examination.	Not stated	Not stated	Students appreciated the knowledge from observing their colleagues. Twelve students developed leadership and clinic management skills via directorships - three student leaders were selected each year.	Not stated	Not stated

Study ID	Student disciplines	Number of students	Student admission to the clinic	Pre-clinic orientation or training	Student outcome measures	Satisfaction	Experience/perceptions	Time spent in the clinic	Other results
**Felder-Heim 2020**	Medicine, Physiotherapy, Dentistry, Pharmacy, Psychology, Care coordination	Not stated	Volunteer	Not stated	Not stated	Not stated	Not stated	Not stated	Not stated
**Fröberg 2018**	Medicine, Nursing, Physiotherapy, Occupational Therapy, Psychology	199	Placement, required	A coordinator introduced students to clinic processes	'Clinical Learning Environment, Supervision and Nurse Teacher evaluation scale'	Majority reported to be satisfied with learning environment, placement structure, relationship with supervisors, and care to patients. Overall high levels of satisfaction with amount and model of supervision and feedback received	Good learning environment mean rating 4.8/5. Overall satisfied with clear philosophy of care outlined to patients, and the individualised care delivered. Clinic concept was valued by all disciplines.	Not stated	Response rate 87%
**Fung 2022**	Medicine, Nursing, Nutrition, Pharmacy, Public Health, Biomedical Sciences	25	Volunteer	A social worker delivered education on geriatric conditions, medication safety, Hong Kong health system, and patient communication skills. Students completed e-	Student feedback survey	92% enjoyed the program: 56% valued its flexibility (56%), 16% thought it was meaningful, 32% reported the program duration was appropriate. 8% did not enjoy any part of the program.	92% felt improved communication skills, 48% felt they could apply class knowledge in the clinic. 16% valued the communication skills and bioethics knowledge. 90% improved knowledge of older people. 96% enjoyed communicating and learning with other	Not stated	Not stated

Study ID	Student disciplines	Number of students	Student admission to the clinic	Pre-clinic orientation or training	Student outcome measures	Satisfaction	Experience/perceptions	Time spent in the clinic	Other results
				learning materials and a knowledge quiz.			students. Communication issues reported: 46% found difficult to communicate only via phone, 20% found difficult to contact participants, 12% found challenging to sustain phone conversations, 44% felt they could not apply class content to the clinic.		
**Garavelis 2023**	Occupational Therapy, Psychology	22	Placement, required	Orientation delivered in week 1 of placement, details not provided	Not stated	Not stated	Not stated	Not stated	Not stated
**Gortney 2018**	Medicine, Social Work, Pharmacy	326	Volunteer	Online orientation guide, an onsite team orientation and a student-delivered health lecture	Not stated	Not stated	Not stated	3 hours	Number of students involved in 24 months: medicine (167, 51.2%), pharmacy (122, 37.4%), and social work (37, 11.4%).

Study ID	Student disciplines	Number of students	Student admission to the clinic	Pre-clinic orientation or training	Student outcome measures	Satisfaction	Experience/perceptions	Time spent in the clinic	Other results
**Henderson-Kalb 2022**	Medicine, Nutrition, Occupational Therapy, Physiotherapy, Social Work, Speech Pathology	171	Placement, required	Trained using assessment tools, learned clinic requirements, watched a 3-minute guiding video and reviewed clinic processes. Pre-clinic team meeting was held.	Written feedback	"Valuable" experience, students enjoyed teamwork.	Students enjoyed learning about the scope of practice of other disciplines, older people, collaboration, and telehealth. A student found difficult to develop rapport with clients via telehealth. Students found easy to transition to telehealth and saw benefit for clients who did not have to commute.	Not stated	Not stated
**Howell 2021**	Dietetics, Nutrition, Kinesiology, Public Health, Health Sciences	25	Placement, elective	Program orientation and training session delivered by the educators	student IPE survey	Most found program very helpful and enjoyed interactions with older adults.	90% agreed that knowledge of roles, communication and teamwork were improved by IPE. 86% improved knowledge of cultural diversity and collaborative skills. Some felt increased confidence in helping older adults. 54% now considered working with geriatric population after graduating. Students wished for a bigger space to deliver intervention. Some felt nervous in presenting	2 weeks	Age range 22-35

Study ID	Student disciplines	Number of students	Student admission to the clinic	Pre-clinic orientation or training	Student outcome measures	Satisfaction	Experience/perceptions	Time spent in the clinic	Other results
							alone and suggested more role-play prior to service delivery.		
**Hu 2016**	Medicine, Nursing, Physiotherapy, Social Work, Pharmacy	Not stated	Volunteer	Not stated	Not stated	Not stated	Not stated	Not stated	Not stated
**Janson 2009**	Medicine, Nursing, Pharmacy	120	Mixed, placement and volunteer	Half day orientation included a 60-minute lecture, a 30-minute clinical discussion, 2.5-hour clinic visits, online course. Training on ICIC model delivered over 2 seminars and small quality improvement projects.	Robert Wood Johnson/ Partners in Quality Education 'Take Care to Learn' Trainee Survey	Not stated	Students demonstrated improvements in all items of ICIC model. At 1 year they felt improved confidence in decision making, using patient information systems and resources in the community. Students felt improved skill in delivering care and education to patients with diabetes.	10 weeks to 2 years	Not stated
**Johnston 2019**	Medicine, Pharmacy	Not stated	Not stated	Not stated	Not stated	Not stated	Not stated	Not stated	Not stated
**Johnston 2020**	Medicine, Pharmacy	Not stated	Not stated	Not stated	Not stated	Not stated	Not stated	Not stated	Not stated
**Kahkoska 2018**	Medicine, Nursing, Physician Assistant, Pharmacy	Not stated	Placement, required	Prior to each clinic, student teams were oriented to clinic processes.	Not stated	Not stated	Not stated	Not stated	Not stated

Study ID	Student disciplines	Number of students	Student admission to the clinic	Pre-clinic orientation or training	Student outcome measures	Satisfaction	Experience/perceptions	Time spent in the clinic	Other results
**Kent 2013**	Medicine, Nursing, Physiotherapy, Occupational Therapy, Dietetics, Nutrition, Social Work	18	Volunteer	Students were given a guiding screening tool.	Not stated	Not stated	Not stated	Not stated	Not stated
**Kent 2016**	Nursing, Physiotherapy, Occupational Therapy, Speech Pathology, Dietetics, Social Work, Pharmacy, Psychology, Podiatry	Not stated	Volunteer	Not stated	Not stated	Not stated	Not stated	Not stated	Not stated
**Krout 2010**	Physiotherapy, Occupational Therapy, Speech Pathology, Recreational Therapy, Audiology	225	Placement, elective and required	Service-learning projects were prepared by students and faculty, several planning meetings between students and elders	Pre-post-test civic engagement questions and satisfaction survey	Very high degrees of satisfaction with program, all survey questions achieved > 79% agreement with positive statements. 92% pleased with service-learning experience.	91.3% improved attitudes towards older adults. 79% thought the amount of time with elders was adequate. 98% valued the educational experience and relevance to their course. 95% improved understanding of a service-learning activity. 10% had difficulties with transportation to	10 hours to a semester	Not stated

Study ID	Student disciplines	Number of students	Student admission to the clinic	Pre-clinic orientation or training	Student outcome measures	Satisfaction	Experience/perceptions	Time spent in the clinic	Other results
							placement site. 90% improved knowledge of older people. Most felt improved communication skills (97%) and teamwork (96%).		
**Lawrence 2015**	Medicine, Nursing	29	Volunteer	Four hours of training in 2 sessions: orientation to the clinic philosophy, processes and how to present info to educators.	Not stated	Not stated	Not stated	Not stated	Not stated
**Leung 2012**	Medicine, Nursing, Pharmacy	11	Volunteer	Two hours of interactive training with physicians, nurses, and psychologists	Not stated	Not stated	Some frustration reported with not finding an appropriate time to call patient, finding it difficult to communicate in a way patient could understand, challenging goal setting, and getting patient to implement changes.	3-6 months	Not stated
**Liang En 2011**	Medicine, Nursing	274	Volunteer	Not stated	Modified FIPSE survey Instrument and the Ability Scale	94% to 100% reported improvement in all nine learning domains, except the ability to make	Nursing students were more likely to report improved leadership skills. Medical students felt improved leadership, teamwork,	Not stated	Ability Scale: Leadership Communication Teamwork Critical thinking

Study ID	Student disciplines	Number of students	Student admission to the clinic	Pre-clinic orientation or training	Student outcome measures	Satisfaction	Experience/perceptions	Time spent in the clinic	Other results
						a clinical diagnosis, for which 79% reported gains.	communication skills, critical thinking, and identification of social problems.		Knowledge n of social issues<br>Ability to take action and take on new responsibilities<br>Ability to see consequences<br>Knowledge acquisition and application
**Meek 2013**	Medicine, Nursing	20	Volunteer	Students oriented on the first placement day; no details provided.	Not stated	Not stated	Not stated	2 weeks	Not stated
**Meuser 2022**	Occupational Therapy, Social Work, Osteopathic Medicine	54	Volunteer	Weekly planning sessions with educators and older adult advisors	Email invitation to identify lessons learned	Students felt satisfied with online sessions being delivered according to plan.	Students felt surprised with older adults' knowledge of computer technology and their willingness to join discussions. Students understood the importance of building rapport and making everyone feel included. They valued planning time prior to zoom sessions, improved perceptions towards older adults and appreciation for their own young age, and the impact of the pandemic	Not stated	Not stated

Study ID	Student disciplines	Number of students	Student admission to the clinic	Pre-clinic orientation or training	Student outcome measures	Satisfaction	Experience/perceptions	Time spent in the clinic	Other results
							on older adults. Students felt anxious prior to online sessions due to assumptions clients would have low motivation.		
**Ng 2020**	Medicine, Nursing, Pharmacy	226	Volunteer	Orientation completed together with secondary school students to prepare for visits, lectures and workshops on program content and in communication difficulties, role-play, mobility aids workshops and caregiving skills	‘Kogan's Attitude towards Old People’, PFAQ and FIPSE	Most felt more prepared to practice as a result of program. 92.4% improved knowledge of health problems in older clients, 92% recommend the program to colleagues	‘Kogan's Attitude towards Old People’ Scale: statistically significant mean increase of 12.8 - reduced ageist attitudes. Most felt improved knowledge and preparation to practice. 92% found IP practice important in patient care. Most improved confidence in caregiving skills and were more aware of problems associated with older age. 90-100% reported improved knowledge in all FIPSE domains, but not in ability to diagnose (81.6%) and to apply knowledge in home visits (80.1%).	6 months	64.6% had previous IP education experience. 62.4% female Median Age 21 (18-41) Number of respondents 181

Study ID	Student disciplines	Number of students	Student admission to the clinic	Pre-clinic orientation or training	Student outcome measures	Satisfaction	Experience/perceptions	Time spent in the clinic	Other results
**Ouyang 2013**	Medicine, Nursing, Physician Assistant, Physiotherapy, Dentistry, Social Work, Pharmacy, Public Health	Not stated	Volunteer, required to attend an elective subject prior	10 hours of elective, 2-hour orientation in communication and interpreter processes, group training led by student clinic coordinator	Not stated	Not stated	Not stated	Not stated	Not stated
**Palma 2020**	Medicine, Nursing, Physician Assistant	Not stated	Volunteer	Not stated	Not stated	Not stated	Not stated	Not stated	Not stated
**Peluso 2014**	Medicine, Nursing, Physician Assistant, Pharmacy	Not stated	Volunteer	Students were trained in clinical knowledge and skills in LTBI management, including lectures, case discussions, practical sessions and documentation skills	Not stated	Not stated	Not stated	Not stated	Not stated
**Reumerman 2021**	Medicine, Nursing, Physician Assistant, Pharmacy	34	Mixed, placement and volunteer	Use of Prescribing Optimalisation Method and START-STOPP criteria. Weekly education/training session.	Satisfaction and learning outcomes via digital student survey	91% found IP program valuable. Out of 100, students reached a medium of 85 in the satisfaction survey.	All agreed IP knowledge improved. Real medication review scenarios were found educational and more interesting than simulation, although working with real patients was perceived	1 week	9 students (29.4%) previous IP education experience. All agreed program would stimulate

Study ID	Student disciplines	Number of students	Student admission to the clinic	Pre-clinic orientation or training	Student outcome measures	Satisfaction	Experience/perceptions	Time spent in the clinic	Other results
							as more difficult. Students developed knowledge of each other's competencies, boundaries, and responsibilities. Pharmacy students less empathetic. Medical students best clinical knowledge but less concerned about medication safety. Pharmacy, nursing and physician assistant students most practical, caring and best knowledge of guidelines.		future IP teamwork.
**Reumerman 2022**	Medicine, Nursing, Pharmacy	Not stated	Mixed, placement and volunteer	Use of Prescribing Optimalization Method, START-STOPP criteria, and medication trigger list	Not stated	Not stated	Not stated	Not stated	Not stated
**Rock 2014**	Medicine, Nursing, Social Work, Law	Not stated	Placement, required	Preparatory classes conducted during the first 9 months of medical school. During first year of visitation, weekly didactic sessions	Not stated	Not stated	Not stated	3.5 years	Not stated

Study ID	Student disciplines	Number of students	Student admission to the clinic	Pre-clinic orientation or training	Student outcome measures	Satisfaction	Experience/perceptions	Time spent in the clinic	Other results
				led by supervising faculty physicians.					
**Rowe 2021**	Nursing, Social Work, Pharmacy	Not stated	Placement, required	Half-day orientation: IPE Core Competencies, roles, clinic demographics, diabetes, depression, team building, and a simulated standardized patient encounter. Educators modelled IP behaviours to students.	Not stated	Not stated	Not stated	Not stated	Not stated
**Sargison 2021**	Occupational Therapy, Speech pathology	11	Placement, required	Comprehensive classroom observations at commencement of placement, regular pre-session check in with early childhood educators	Not stated	Not stated	Not stated	10-12 weeks	Not stated

Study ID	Student disciplines	Number of students	Student admission to the clinic	Pre-clinic orientation or training	Student outcome measures	Satisfaction	Experience/perceptions	Time spent in the clinic	Other results
**Sarovich 2022**	Occupational Therapy, Physiotherapy, Speech Pathology, Dietetics, and Social Work	Not stated	Placement, required	Not stated	Not stated	Not stated	Not stated	Not stated	Not stated
**Sealey 2017**	Medicine, Nursing, Physiotherapy, Exercise Physiology, Speech Pathology, Dentistry, Pharmacy, Psychology	18	Mixed, placement and volunteer	Induction and help with designing education session	Health professions knowledge questionnaire, RIPLS, focus groups	Students enjoyed the interdisciplinary experience and working in a new environment. All students would recommend to others.	RIPLS: statistical significance in two questions, demonstrating improved understanding IP education and own role in health care. Students reported limited understanding of one another's role, which was developed during the program. Improved self-confidence, group speaking, teamwork and leadership skills were reported. They felt improved communication and appreciated getting real time feedback.	At least 3 sessions	Not stated
**Seymour 2010**	Nursing, Physiotherapy,	7	Volunteer	Several education sessions given by program director	Student reflection questionnaire	Not stated	All felt improved communication. Some improved ability to	21 months	Not stated

Study ID	Student disciplines	Number of students	Student admission to the clinic	Pre-clinic orientation or training	Student outcome measures	Satisfaction	Experience/perceptions	Time spent in the clinic	Other results
	Exercise Physiology			and coordinator on chronic diseases, reading discharge summaries, facilitation of social interactions, review of Health Promotions Inventory			think globally. Students improved knowledge of a holistic approach, health problems faced by older people, other health professional skills, and improved awareness of lack of resources for this age group. They had greater appreciation for the complex nature of disabilities. Support from coordinator was reassuring and helpful. Improved interpersonal skills and rapport building facilitated health promotion advocacy. Some reported frustration communicating with elders with multiple health concerns.		
**Shekar 2020**	Medicine, Nursing, Physician Assistant, Social Work	Not stated	Volunteer	One hour training session: curricular content and teaching strategies by paediatric resident physician at the Children's Hospital	Not stated	Not stated	Not stated	Not stated	Not stated

Study ID	Student disciplines	Number of students	Student admission to the clinic	Pre-clinic orientation or training	Student outcome measures	Satisfaction	Experience/perceptions	Time spent in the clinic	Other results
**Sheu 2010**	Medicine, Nursing, Dentistry, Pharmacy	477	Placement, elective	Two clinical skills training sessions. Months of writing grants and liaising with key partners prior to starting the 2 permanent clinics. 15-25 students worked together on a weekly basis.	Student participation rates and annual enrolment	Not stated	Not stated	1-3 clinics	54% pharmacy, 24% medicine, 17% nursing, 5% dentistry students. 93% first years. 2008-2009: average participation in 1.89 clinics (SD 1.32). Annual student enrolment up from 67 in 2004/5 to 142 in 2008/9.
**Sultan 2022**	Medicine, Nursing, Physician Assistant, Pharmacy	Not stated	Placement, required	Not stated	Not stated	Not stated	Not stated	Not stated	Not stated
**Virtue 2018**	Dentistry, Pharmacy	Not stated	Not stated	Not stated	Not stated	Not stated	Not stated	Not stated	Not stated
**Walker 2022**	Nursing, Physiotherapy, Exercise Physiology, Dietetics, Social Work, Psychology	14	Placement, elective	Skill building sessions to learn use of video conferencing equipment and telehealth	Yarning sessions, observations and semi-structured interviews	Not stated	Students perceived a need to improve communications skills and knowledge of technology. Surprised with ease of delivering a program online. They appreciated telehealth allowed communication during the pandemic.	3 to 17 weeks	Not stated

Study ID	Student disciplines	Number of students	Student admission to the clinic	Pre-clinic orientation or training	Student outcome measures	Satisfaction	Experience/perceptions	Time spent in the clinic	Other results
							They valued knowledge and skills learned using different online platforms, particularly with rural clients. They valued client motivation and engagement. Some students found difficult to use telephone to deliver intervention. Students wished they learned about telehealth delivery during their course to help with preparing to placement.		

Key: FIPSE: Fund for the Improvement of Post-Secondary Education, ICIC: Improving Chronic Illness Care, IP: interprofessional, IPE: interprofessional education, LTBI: latent tuberculosis infection, PFAQ: Palmore Facts on Aging Quiz, RIPLS: Readiness for Interprofessional Learning Scale

Article title: Impact of interprofessional student led health clinics for patients, students and educators: a scoping review
Journal name: Advances in Health Sciences Education
Author names: Janine Prestes Vargas, Moira Smith, Lucy Chipchase, Meg E. Morris
Affiliation of corresponding author: Victorian Rehabilitation Centre, Glen Waverley, and ARCH and CERI La Trobe University
Email of corresponding author: m.morris@latrobe.edu.au
