# Supplementary material for: Impact of interprofessional student led health clinics for patients, students and educators: a scoping review
Source: Adv Health Sci Educ Theory Pract. 2024 Jun 6;30(1):321–45. doi: 10.1007/s10459-024-10342-2 (PMC11925975; doi:10.1007/s10459-024-10342-2)
Supplement: Supplementary file 6 — Supplementary Material 6 [file 10459_2024_10342_MOESM6_ESM.pdf]

SUPPLEMENTARY MATERIAL 6: CLINICAL EDUCATOR OUTCOMES

Study ID	Educator disciplines	Number of educators	Outcome measures	Experience/Perceptions	Amount of supervision provided	Type of supervision	Other
**Beckman 2022**	Nursing, Physiotherapy, Exercise Physiology, Psychology	5	Surveys, semi-structured interviews	80% felt capable of providing interprofessional education to students. After placement, 20% more educators thought students were prepared for interprofessional care. Theme 1: strategies to support interprofessional education: value of modelling to enhance student competence, IP setting important to student IP learning, understanding of own role increased student confidence. Theme 2: challenges in the delivery of interprofessional education: placing students together in teams was challenging due to distinct student timetables and placement periods. Feedback and supervision were challenged by limited time observing students, and student assessment was difficult due to the large number of students, so educators had to prioritise which students to observe.	Full time supervision	Inter professional	Postgraduate practice experience: <2 years (20%, n = 1), 2-6 years (40%, n = 2), >10 years (40%, n = 2).
**Bird 2022**	Occupational therapy, Speech Pathology	4	Semi-structured interviews	Supervisors recognised students improved communication skills, and the benefit of a co-created service for student learning, as students immersed themselves in the culture to empower and improve health. Orientation was perceived as important in creating opportunities for student IP work. Ongoing communication and support facilitated student learning, confidence and understanding of a culturally safe service. Supervisors observed student IP knowledge growth improved collaboration and respect towards each other's role and towards visiting health professionals. Ongoing encouragement facilitated student confidence to deal with cultural and geographical challenges. Students improved bonding with all stakeholders over time, which resulted in improved recognition of older person's needs. Students were growing as they learned to co-create a service, without controlling but fitting in with the culture and practices, and offered flexible and accessible suggestions for	Full time onsite and remote supervision	Inter professional and single discipline	

Study ID	Educator disciplines	Number of educators	Outcome measures	Experience/Perceptions	Amount of supervision provided	Type of supervision	Other
				improvement. Yolŋu cultural consultants valued supervisor prior knowledge of the aboriginal community to support and guide students onsite full time.<br>Service manager valued co-design skills offered by supervisors and students and how service naturally evolved with time, they also valued resources left by students, and the skills in addressing community needs.			
**Fröberg 2018**	Medicine, Nursing, Physiotherapy, Occupational Therapy, Psychology	35	Semi-structured interviews	Theme 1: Organization, structure and administration: The support and time given to supervisors by the clinic facilitated student-centred supervision, feedback and assessment, including the identification of different supervisory styles. Separate rooms for students and supervisors were perceived as a facilitator to student autonomy. Clinic promoted interprofessional collaboration between students and supervisors. Theme 2: tuition as a pedagogical entity: supervising in the clinic was perceived as more pedagogical than in everyday practice. Supervisors had to adapt to new focus of being the supervisor and the caregiver. Longer time in sessions was perceived as adequate for student learning. Theme 3: Control over provided care: difficulty finding balance between student autonomy and supervisor control, and importance of trusting the student. Theme 4: Reflection on professional and pedagogical competence: supervisors reflected on the importance of further developing their clinical supervision skills and attending continuous professional development, some felt a lack of skills to deliver supervision and assessment at the clinic.	Medical, nursing and psychology students: 1 supervisor per 2 students. Physiotherapy and occupational therapy: 1 supervisor per student	Inter professional, Single discipline	Not stated

Article title: Impact of interprofessional student led health clinics for patients, students and educators: a scoping review
Journal name: Advances in Health Sciences Education
Author names: Janine Prestes Vargas, Moira Smith, Lucy Chipchase, Meg E. Morris
Affiliation of corresponding author: Victorian Rehabilitation Centre, Glen Waverley, and ARCH and CERI La Trobe University
Email of corresponding author: m.morris@latrobe.edu.au
